# Impact of Qi-Invigorating Traditional Chinese Medicines on Diffuse Large B Cell Lymphoma Based on Network Pharmacology and Experimental Validation

**DOI:** 10.3389/fphar.2021.787816

**Published:** 2021-12-09

**Authors:** Qian Huang, Jinkun Lin, Surong Huang, Jianzhen Shen

**Affiliations:** Fujian Institute of Hematology, Fujian Medical Center of Hematology, Fujian Provincial Key Laboratory of Hematology, Fujian Medical University Union Hospital, Fuzhou, China

**Keywords:** qi-invigorating herbs, traditional Chinese medicine, diffuse large B cell lymphoma, network pharmacology, tumor microenvironment

## Abstract

**Background:** It has been verified that deficiency of Qi, a fundamental substance supporting daily activities according to the Traditional Chinese Medicine theory, is an important symptom of cancer. Qi-invigorating herbs can inhibit cancer development through promoting apoptosis and improving cancer microenvironment. In this study, we explored the potential mechanisms of Qi-invigorating herbs in diffuse large B cell lymphoma (DLBCL) through network pharmacology and *in vitro* experiment.

**Methods:** Active ingredients of Qi-invigorating herbs were predicted from the Traditional Chinese Medicine Systems Pharmacology Database. Potential targets were obtained via the SwissTargetPrediction and STITCH databases. Target genes of DLBCL were obtained through the PubMed, the gene-disease associations and the Malacards databases. Overlapping genes between DLBCL and each Qi-invigorating herb were collected. Hub genes were subsequently screened via Cytoscape. The Gene Ontology and pathway enrichment analyses were performed using the DAVID database. Molecular docking was performed among active ingredients and hub genes. Hub genes linked with survival and tumor microenvironment were analyzed through the GEPIA 2.0 and TIMER 2.0 databases, respectively. Additionally, *in vitro* experiment was performed to verify the roles of common hub genes.

**Results:** Through data mining, 14, 4, 22, 22, 35, 2, 36 genes were filtered as targets of Ginseng Radix et Rhizoma, Panacis Quinquefolii Radix, Codonopsis Radix, Pseudostellariae Radix, Astragali Radix, Dioscoreae Rhizoma, Glycyrrhizae Radix et Rhizoma for DLBCL treatment, respectively. Then besides Panacis Quinquefolii Radix and Dioscoreae Rhizoma, 1,14, 10, 14,13 hub genes were selected, respectively. Molecular docking studies indicated that active ingredients could stably bind to the pockets of hub proteins. CASP3, CDK1, AKT1 and MAPK3 were predicted as common hub genes. However, through experimental verification, only CASP3 was considered as the common target of Qi-invigorating herbs on DLBCL apoptosis. Furthermore, the TIMER2.0 database showed that Qi-invigorating herbs might act on DLBCL microenvironment through their target genes. Tumor-associated neutrophils may be main target cells of DLBCL treated by Qi-invigorating herbs.

**Conclusion:** Our results support the effects of Qi-invigorating herbs on DLBCL. Hub genes and immune infiltrating cells provided the molecular basis for each Qi-invigorating herb acting on DLBCL.

## Introduction

Diffuse large B cell lymphoma (DLBCL) is an aggressive lymphoma and the most common subtype of lymphoma, accounting for more than 30% of the lymphoma cases (Siegel et al., 2021). Although 60–70% of DLBCL patients reach long-term remission in response to a combination of rituximab, cyclophosphamide, doxorubicin, and prednisone (R-CHOP) immunochemotherapy, tumor recurrent, coupled with therapeutic resistant figures remain high.

In recent years, several agents constitute the new landscape of treatments for DLBCL, including brentuximab vedotin (Svoboda et al., 2021), ibrutinib (Younes et al., 2019) and polatuzumab vedotin (Sehn et al., 2020). Besides, targeting tumor microenvironment (TME) is one promising option for increasing evidence strongly highlights that lymphomagenesis and DLBCL progression mainly rely on its microenvironment (Scott and Gascoyne, 2014).

Immune cells are main components of TME surrounding DLBCL cells, including T lymphocytes, macrophages and natural killer (NK) cells. It has been identified that a heterogeneous DLBCL microenvironment differs significantly in the subtype of tumor-infiltrating immune cells. For instance, a high proportion of programmed cell death protein 1 (PD1) + CD8^+^ T cells and programmed cell death-ligand 1 (PD-L1) + T cells in the TME was found to predict poor survival in DLBCL, whereas high expression of immune checkpoint cytotoxic T-lymphocyte-associated protein 4 (CTLA4) on T cells might be associated with favorable outcome (Xu-Monette et al., 2019). A recent study indicated that high proportions of T-cell immunoglobulin, mucin-domain containing 3 (TIM3) + T cells and TIM3+CD4^+^ cells have an independent adverse impact on survival (Autio et al., 2021). However, the above targeted therapy is not always feasible in DLBCL (Ansell et al., 2019; Barrueto et al., 2020; Kline et al., 2020). Much is still unknown concerning DLBCL microenvironment. Therefore, there is an urgent need to develop alternative or complementary therapeutics for DLBCL.

Traditional Chinese medicine (TCM) is gradually being accepted as a therapeutic option of cancer for they have provided much promise *in vivo* experiment and clinical trials (Parekh et al., 2009; Xiang et al., 2019). Based on TCM theory, the human body is recognized as an organic entity with Yin-Yang balance, which is a philosophical concept that represent a unity of opposing forces in the Universe. Generally speaking, Yin refers to stable or negative factors, while Yang represents positive and active features. if the Yin-Yang balance in a single individual is broken, the human body becomes sick. This theory is somewhat similar to TME. Qi is one of the most important products derived from the interaction between Yin and Yang, which stimulates the flow of blood throughout the body, and promotes the absorption and utility of the nutrients of food. The deprivation of Qi can be caused by many factors including malnutrition, fatigue, surgery and chronic diseases, leading to symptoms such as cough, short breath, muscle weakness, and immune-deficiency. Previous studies reported qi deficiency was an important syndrome of cancer (Hou et al., 2021; Qi et al., 2021). Qi-invigorating herbs, including Ginseng Radix et Rhizoma, Astragali Radix, and Glycyrrhizae Radix et Rhizoma, have been verified for their positive roles in cancer immune regulation (Jiang et al., 2019; Wang et al., 2020a). Several Qi-invigorating decoctions can inhibit cancer development through altering cancer immune microenvironment *in vitro* and vivo (Du et al., 2016; Wu et al., 2019; Xu et al., 2020). Furthermore, some Qi-invigorating herbs could directly promote the apoptosis of tumor cells *in vivo* and vitro (Xiong et al., 2018; Wang et al., 2020b). In lymphoma, TCMs have been verified to enhance physical function, reduce adverse effects of chemotherapy, and improve long-term survival (Hijikata et al., 1995; Ben-Arye et al., 2010; Guo et al., 2015; Kim et al., 2018). However, the underlying mechanisms and potential targets of Qi-invigorating herbs in DLBCL remain unclear. It is this idea that is the starting point of this article hoping to provide a theoretical basis for the application of Qi-invigorating herbs in DLBCL.

**FIGURE 1:**
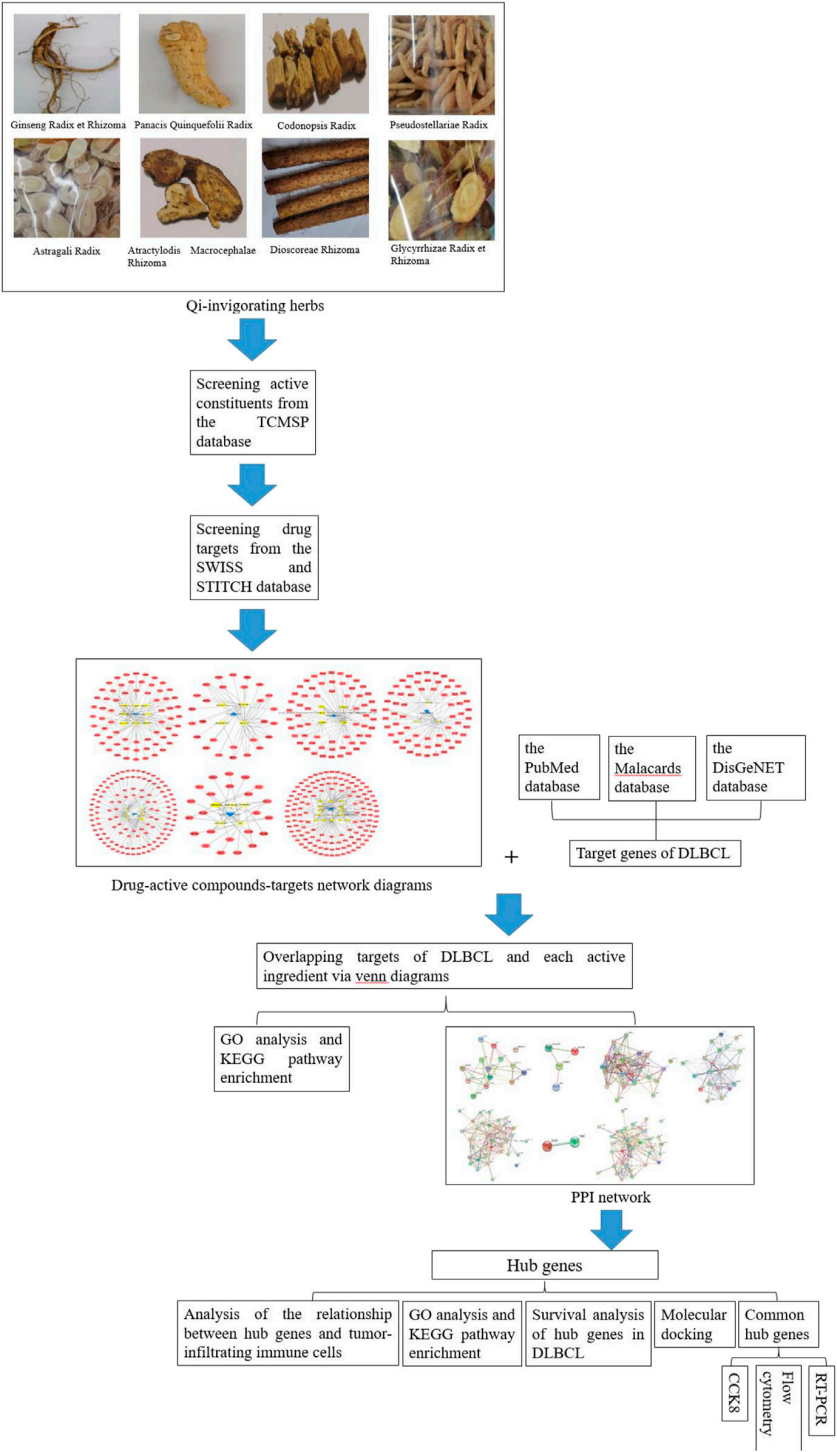
The detailed flowchart of this network pharmacology based study.

In this study, we conducted a network pharmacology strategy for Qi-invigorating herbs. The so-called network pharmacology is a method which combines systems biology with multidirectional pharmacology based on high-throughput omics data analysis and network database retrieval (Hopkins, 2008; Li and Zhang, 2013). Through this discipline and experimental validation, the interaction between Qi-invigorating herbs and DLBCL would be discussed for the first time, which is helpful to elucidate the impact of Qi-invigorating herbs on DLBCL and provide guidance for herb selection, prescription formation, or even new drug design. The detailed flowchart of this study was shown in Figure 1.

## Materials and Methods

### Screening Active Constituents of Qi-Invigorating Herbs

Eight common Qi-invigorating herbs were discussed in this study, including Ginseng Radix et Rhizoma (RenShen), Panacis Quinquefolii Radix (XiYangShen), Codonopsis Radix (DangShen), Pseudostellariae Radix (TaiZiShen), Astragali Radix (HuangQi), Glycyrrhizae Radix et Rhizoma (GanCao), Atractylodis Macrocephalae Rhizoma (BaiZhu) and Dioscoreae Rhizoma (ShanYao). All chemical constituents of the above herbs were obtained from the Traditional Chinese Medicine Systems Pharmacology (TCMSP) database (https://tcmsp-e.com/) (Ru et al., 2014). The potential active ingredients of the above Qi-invigorating herbs were selected according to the oral bioavailability (OB)≥30% (Xu et al., 2012), the Drug-Likeness (DL) Index≥0.18 and the drug half-life (HL)≥4 h (Jin et al., 2021). Chemical structures of the active ingredients were then collected from the PubChem database (https://pubchem.ncbi.nlm.nih.gov/) (Kim et al., 2016).

### Screening Drug Targets

The target genes of the active ingredients were obtained from the SwissTargetPrediction (http://www.swisstargetprediction.ch/) and STITCH (https://stitch.embl.de/) databases (Kuhn et al., 2008; Daina et al., 2019). By uploading chemical structures (SMILE files) to these databases and selecting “*Homo sapiens*” for the species, results were outputted. In the STITCH database, a target with combined-score ≥ 0.7 was selected, while in the SwissTargetPrediction database, probability ≥ 0.7 was assigned as the criteria for screening target genes of the active ingredients. The gene names of the targets were obtained from the UniProt database (https://www.uniprot.org/) (Consortium, 2019). All the target genes of each active ingredient were obtained after combining the two databases and excluding duplicates. The target genes of each Qi-invigorating herb were collected after combining target genes of its active ingredients and excluding the repetitive targets.

### Obtaining Potential Target Genes for Diffuse Large B Cell Lymphoma

We used “diffuse large B cell lymphoma,” “diffuse large-B cell lymphoma” and “DLBCL” as keywords to identify DLBCL-related genes from the PubMed database (update time: September 15, 2021, https://www.ncbi.nlm.nih.gov/gene), the gene-disease associations database (DisGeNET, https://www.disgenet.org/) and the Malacards (https://www.malacards.org/) database (Piñero et al., 2017; Rappaport et al., 2017; Piñero et al., 2020). Only those genes from “*Homo sapiens*” were included. In the end, a total of 1174 target genes of DLBCL were obtained after removing duplicates.

### Constructing Protein-Protein Interaction Networks and Screening Key Targets

Venn diagrams via R software 4.1.0 were conducted to show overlapping targets between DLBCL and each Qi-invigorating herb. These targets were considered as potential targets for Qi-invigorating herbs acting on DLBCL.

To illustrate the enrichment of candidate targets in terms of the gene function, the study applied the Database for Annotation Visualization and Integrated Discovery (DAVID, https://david.ncifcrf.gov/) 6.8 to perform Gene Ontology (GO) and Kyoto Encyclopedia of Genes and Genome (KEGG) analyses (Huang et al., 2007). GO contains molecular function (MF), cellular component (CC), and biological process (BP) analyses. *p*-value < 0.05 were set as the criteria for screening (Huang et al., 2009).

PPI networks of candidate targets were constructed using the STRING database (https://string-db.org/) (Szklarczyk et al., 2021). We also used the Cytoscape 3.7.2 software to construct PPI networks based on the STRING results (Shannon et al., 2003). The criteria for considering significant was interactions with a combined score ≥ 0.7. Genes with degrees ≥ 5 were considered as hub genes. The hub genes were also input into the DAVID 6.8 for GO and KEGG enrichment analyses. These hub genes were subsequently prepared for molecular docking.

### Molecular Docking

The crystal structures of the above hub genes were downloaded from the Protein Data Bank (https://www.pdb.org/) (Berman et al., 2000). PyMol (version 2.3.2) was used to process proteins, including removing water and ligands. The 2D structures of the active ingredients were obtained from the PubChem database (https://pubchem.ncbi.nlm.nih.gov/) and considered as ligands. AutoDockTools 1.5.6 was run for molecular docking (Trott and Olson, 2010). During the docking process, hydrogen atoms were added to all proteins, and partial atomic charges were calculated. Target proteins were held as rigid and ligands allowed traveling freely. After the flexible-ligand docking, the grid box size was set to 40 × 40 × 40 points with a spacing of 0.375 Å. The Lamarckian Genetic Algorithm was used to search for populations of 150 individuals with a mutation rate of 0.02 over 10 generations. The results were sorted on the basis of the binding energy. The smaller the binding energy value, the more stable the structure after binding.

### Analyzing Genes Acting on Diffuse Large B Cell Lymphoma Survival

The GEPIA2 database (http://gepia2.cancer-pku.cn/) was used for validation of hub gene expression in DLBCL and survival analysis (Tang et al., 2019).

### Screening Common Hub Genes and Active Ingredients

A Venn diagram was constructed to select common hub genes and ingredients. Hub genes and targeted active ingredients existed in at least three groups were considered as common hub genes and ingredients, respectively.

### Performing Experimental Verification

#### Preparation of Qi-Invigorating Herbs

All the Qi-invigorating herbs, including Ginseng Radix et Rhizoma, Codonopsis Radix, Pseudostellariae Radix, Astragali Radix, Glycyrrhizae Radix et Rhizoma, were purchased from the TCM pharmacy of Fujian Medical University Union Hospital, Fujian, China. Each herb (10 g) was placed in a beaker, then boiled water (100 ml) was added and the solution was kept boiling for 3 hours (Chen et al., 2019; Miao et al., 2019). The decoctions were concentrated to 0.2 g/ml. The supernatant was collected as research objects after centrifugation, filtration, and sterilization (Chen et al., 2019; Miao et al., 2019).

#### Cell Cultivation

Human DLBCL cell lines SU-DHL-2 (activated B-cell-like lymphoma) and SU-DHL-6 (germinal center B-cell-like lymphoma) were obtained from the American Type Culture Collection (ATCC, Manassas, VA, United States) and cultured in Roswell Park Memorial Institute-1640 (RPMI-1640) medium (HyClone, United States) supplemented with 10% fetal bovine serum (FBS) (CellBox, China) and 1% penicillin-streptomycin. All the cell lines were authenticated using short tandem repeat analysis. Cells were maintained at 37°C in standard culture conditions.

#### Cell Viability Assay

1 × 10^4^ SU-DHL-2 or SU-DHL-6 cells were incubated into the 96‐well plates in 100 μL serum medium per well. A series of two-fold dilutions of decoctions were carried out to give concentrations of 10, 5, 2.5, 1.25, 0.625 and 0.313 mg/ml. Untreated tumor cells and complete medium (blank control) were left as control groups. After 48 h incubation, 10 µL of CCK8 (AbMole Bioscience) was added into all wells and the plates were further incubated for 2 h. Then, the plates were read at 450 and 630 nm as reference wavelength. After background subtraction, half maximal inhibitory concentration (IC50) was calculated using the SPSS 20.0 (IBM, United States). The experiments were repeated in triplicate for each inhibitor concentration.

#### Cell Proliferation Analysis

Proliferative ability of DLBCL cells was analyzed by CCK-8. Briefly, 1 × 10^4^ cells were incubated into the 96‐well plates in 100 μL serum medium. Subsequently, the cells were left negative control and experimental groups for 0, 24, 48, 72 h. The plates were read at 450 and 630 nm as reference wavelength. Each experiment was repeated in triplicate.

#### Flow Cytometry

The Annexin V-APC/propidium iodide (PI) apoptosis detection kit (KeyGEN, China) was used to detect apoptosis by flow cytometry. DLBCL cells were cultured *in vitro* and divided into nine groups, including untreated (blank control), negative control, Astragali Radix group, Glycyrrhizae Radix et Rhizoma group, Ginseng Radix et Rhizoma group, Pseudostellariae Radix group and Codonopsis Radix group. After cultivation for 48 h, cells were washed with PBS, resuspended in 100 µL binding buffer at a concentration of 2 × 10^6^ cells/ml, and anti-Annexin V APC-conjugated antibody and PI were added. The mixtures were incubated for 15 min at room temperature, supplemented with binding buffer to 500 µL and processed by BD Accuri C6 Plus Flow Cytometry (BD Biosciences, United States). Data were analyzed through flow cytometry (BD PharmingenTM, United States).

#### Quantitative Real-Time Polymerase Chain Reaction

Total RNA of cells was extracted by TRIzol (CWBIO, China) and sequentially purified via the chloroform, isopropanol, and 70% ethanol. Reverse transcription for mRNA relied on a kit named Hifair III 1^st^ Strand cDNA Synthesis Supermix for qPCR (YEASEN, China). qRT-PCR was carried out on Applied Biosystems 7500/7500 Fast Real-time PCR System (Thermo Fisher Scientific, United States) and QuantStudio™ 5 Real-Time PCR Instrument (384-well block, Thermo Fisher Scientific, United States). The GADPH was used to normalize mRNA samples. Relative quantification of target primers was calculated by the 2-ΔΔCT method. The experiment was repeated three times. Primers used for qRT-PCR analysis are listed as follow: GADPH Forward (5′-3′) TTG​GTA​TCG​TGG​AAG​GAC​TCA, Reverse (5′-3′) AGT​AGA​GGC​AGG​GAT​GAT​GTT; CASP3 Forward (5′-3′) CAT​GGA​AGC​GAA​TCA​ATG​GAC​T, Reverse (5′-3′) CTG​TAC​CAG​ACC​GAG​ATG​TCA; CDK1 Forward (5′-3′) GTC​AGT​CTT​CAG​GAT​GTG​CT, Reverse (5′-3′) ATG​TAC​TGA​CCA​GGA​GGG​AT; AKT1 Forward (5′-3′) GTC​ATC​GAA​CGC​ACC​TTC​CAT, Reverse (5′-3′) AGC​TTC​AGG​TAC​TCA​AAC​TCG​T; MAPK3 Forward (5′-3′) GCT​TCC​GCC​ATG​AGA​ATG​TC; Reverse (5′-3′) GGC​GGA​GTG​GAT​GTA​CTT​GA.

### Analyzing Genes Acting on Diffuse Large B Cell Lymphoma Tumor Microenvironment

The TIMER database (http://timer.cistrome.org/) is a database for comprehensive analysis of immune infiltrating cells across multiple cancer types. We applied TIMER2.0 to study the relationship between hub genes and tumor-infiltrating immune cells [B cells, CD4^+^ T cells, CD8^+^ T cells, neutrophils, macrophages, and myeloid dendritic cells (DCs), myeloid derived suppressor cells (MDSC)] in DLBCL (Li et al., 2020).

### Statistical Analysis

All data were analyzed by the SPSS 20.0 (IBM, United States) or GraphPad Prism 7 (GraphPad Software Inc., United States) software. *p* < 0.05 was considered as statistical significance. *p* < 0.01 was considered as obvious statistical significance.

## Results

### Filtering of Active Constituents of Qi-Invigorating Herbs

Among Qi-invigorating herbs, 20 active ingredients were identified from Ginseng Radix et Rhizoma, 9 from Panacis Quinquefolii Radix, 19 from Codonopsis Radix, 6 from Pseudostellariae Radix, 16 from Astragali Radix, 5 from Atractylodis Macrocephalae Rhizoma, 16 from Dioscoreae Rhizoma and 76 from Glycyrrhizae Radix et Rhizoma. The details of active ingredients were summarized in Supplementary Table S1.

### Screening of Drug Targets

**FIGURE 2:**
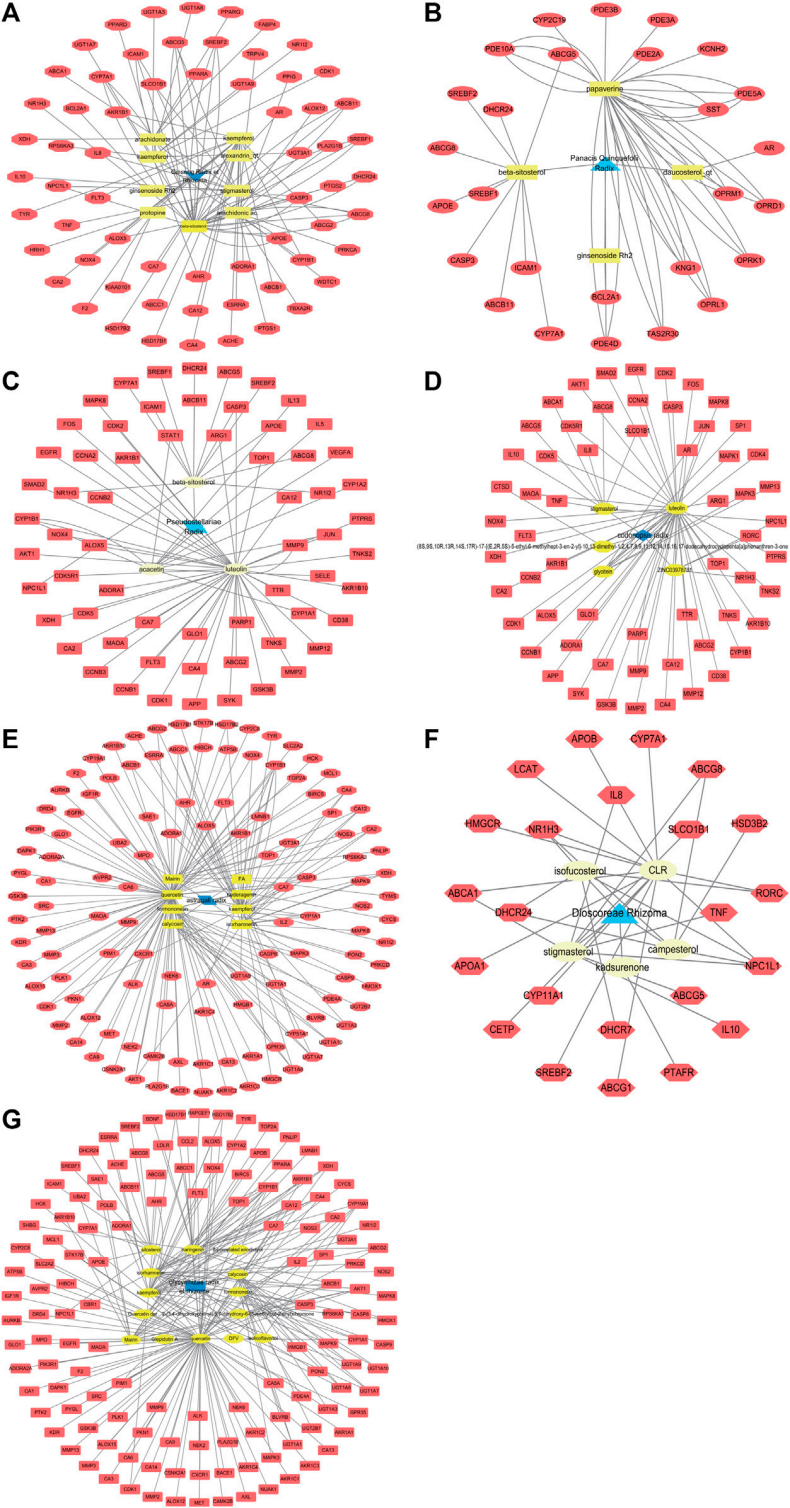
Network diagrams of drug-active ingredients-targets. The red nodes represent target genes, the yellow nodes represent active ingredients, while the blue nodes mean herb. **(A)**. The network diagram of Ginseng Radix et Rhizoma-active ingredients-targets; **(B)**. The network diagram of Panacis Quinquefolii Radix-active ingredients-targets; **(C)**. The network diagram of Pseudostellariae Radix-active ingredients-targets; **(D)**. The network diagram of Codonopsis Radix-active ingredients-targets; **(E)**. The network diagram of Astragali Radix-active ingredients-targets; **(F)**. The network diagram of Dioscoreae Rhizoma-active ingredients-targets; **(G)**. The network diagram of Glycyrrhizae Radix et Rhizoma-active ingredients-targets.

Through the SwissTargetPrediction and Stitch platforms, 63 genes were selected as candidate targets of Ginseng Radix et Rhizoma, 27 genes as candidate targets of Panacis Quinquefolii Radix, 62 genes as candidate targets of Codonopsis Radix, 63 genes as candidate targets of Pseudostellariae Radix, 121 genes as candidate targets of Astragali Radix, 0 genes as candidate targets of Atractylodis Macrocephalae Rhizoma, 23 genes as candidate targets of Dioscoreae Rhizoma, and 136 genes as candidate targets of Glycyrrhizae Radix et Rhizoma. The drug-active compounds-targets network diagrams were shown in Figure 2 and the target names of Qi-invigorating herbs were summarized in Supplementary Table S2.

### Screening of Common Targets Between Qi-Invigorating Herbs and Diffuse Large B Cell Lymphoma

A total of 1174 target genes associated with DLBCL were retrieved from the PubMed, DisGeNET, and Malacards databases. Common targets of DLBCL and each Qi-invigorating herb were considered as interacting targets between herbs and DLBCL. As shown in Table 1, there existed 14 interacting targets between Ginseng Radix et Rhizoma and DLBCL, 4 targets between Panacis Quinquefolii Radix and DLBCL, 22 targets between Codonopsis Radix and DLBCL, 22 targets between Pseudostellariae Radix and DLBCL, 35 targets between Astragali Radix and DLBCL, 2 targets between Dioscoreae Rhizoma and DLBCL, and 36 targets between Glycyrrhizae Radix et Rhizoma and DLBCL.

**TABLE 1:** Interacting targets between DLBCL and Qi-invigorating herbs.

Herbs	Interacting Targets
Ginseng Radix et Rhizoma	CDK1 ABCB1 AHR TNF PTGS2 BCL2A1 CASP3 ABCG2 IL10 ICAM1 ABCC1 AR PRKCA RPS6KA3
Panacis Quinquefolii Radix	BCL2A1 CASP3 ICAM1 AR
Codonopsis Radix	CCNB1 CD38 CDK1 TNF CDK4 CCNA2 CASP3 PARP1 ABCG2 IL10 MAPK1 SYK FOS MAPK3 CDK5R1 CDK2 CDK5 AKT1 JUN AR APP TOP1
Pseudostellariae Radix	CCNB1 CD38 CDK1 CYP1A1 IL5 VEGFA CCNA2 IL13 STAT1 CASP3 PARP1 ABCG2 SYK FOS ICAM1 CDK5R1 CDK2 CDK5 AKT1 JUN APP TOP1
Astragali Radix	NOS2 MET UGT1A1 BIRC5 PLK1 ALK CDK1 CYP1A1 CASP8 ABCB1 AHR PIM1 HCK AURKB TYMS MCL1 CASP3 KDR ABCG2 HMOX1 LMNB1 IL2 TOP2A IGF1R MAPK3 CASP9 AKT1 ABCC1 AR DAPK1 NEK2 PDE4A RPS6KA3 HMGB1 TOP1
Dioscoreae Rhizoma	TNF IL10
Glycyrrhizae Radix et Rhizoma	NOS2 MET UGT1A1 BIRC5 PLK1 ALK BDNF CDK1 CYP1A1 CASP8 ABCB1 AHR PIM1 HCK AURKB MCL1 CCL2 CASP3 KDR ABCG2 HMOX1 LMNB1 IL2 TOP2A IGF1R ICAM1 MAPK3 CASP9 AKT1 ABCC1 DAPK1 NEK2 PDE4A RPS6KA3 HMGB1 TOP1

### Gene Ontology and Pathway Functional Enrichment Analyses of Interacting Targets

To further explore functions of the above interacting targets, GO and pathway functional enrichment analyses were performed with DAVID Bioinformatics Resources 6.8. It was demonstrated that there were some similarities as well as differences among Qi-invigorating herbs. According to *p*-values, the top 5 BP, MF, CC and KEGG pathways of interacting targets were shown in Table 2. For instance, interacting targets between Ginseng Radix et Rhizoma and DLBCL had main KEGG pathways in African trypanosomiasis, NF-kappa B signaling pathway, amoebiasis, TNF signaling pathway, natural killer cell mediated cytotoxicity; while interacting targets between Panacis Quinquefolii Radix and DLBCL had main pathways in viral myocarditis, NF-kappa B signaling pathway, TNF signaling pathway; interacting targets between Codonopsis Radix and DLBCL had main pathways in hepatitis B, T cell receptor signaling pathway, pertussis, viral carcinogenesis, progesterone-mediated oocyte maturation; interacting targets between Pseudostellariae Radix and DLBCL had main pathways in hepatitis B, Epstein-Barr virus infection, TNF signaling pathway, pathways in cancer, osteoclast differentiation; interacting targets between Astragali Radix and DLBCL had main pathways in pathways in cancer, progesterone-mediated oocyte maturation, toxoplasmosis, oocyte meiosis, colorectal cancer; interacting targets between Dioscoreae Rhizoma and DLBCL had main pathways in asthma, African trypanosomiasis, allograft rejection, malaria, inflammatory bowel disease; interacting targets between Glycyrrhizae Radix et Rhizoma and DLBCL had main pathways in pathways in cancer, progesterone-mediated oocyte maturation, chagas disease, TNF signaling pathway, toxoplasmosis.

**TABLE 2:** GO and signaling pathway analysis of common targets between Qi-invigorating herbs and DLBCL.

Herbs	Type	Term	Function	Count	*p* value
Ginseng Radix et Rhizoma	Biological Process	GO:0042493	response to drug	8	9.30E-10
		GO:0051384	response to glucocorticoid	4	1.54E-05
		GO:0043066	negative regulation of apoptotic process	5	3.13E-04
		GO:0045429	positive regulation of nitric oxide biosynthetic process	3	4.91E-04
		GO:0006810	transport	4	0.002163
	Cellular Component	GO:0045121	membrane raft	3	0.009137
		GO:0005886	plasma membrane	8	0.014002
		GO:0005654	nucleoplasm	6	0.036623
		GO:0005737	cytoplasm	8	0.049495
	Molecular Function	GO:0042626	ATPase activity, coupled to transmembrane movement of substances	3	5.09E-04
		GO:0005515	protein binding	13	0.002657
		GO:0005524	ATP binding	6	0.0038
		GO:0008559	xenobiotic-transporting ATPase activity	2	0.003845
		GO:0005215	transporter activity	3	0.01019
	KEGG pathway	hsa05143	African trypanosomiasis	4	2.15E-05
		hsa04064	NF-kappa B signaling pathway	4	3.96E-04
		hsa05146	Amoebiasis	4	7.07E-04
		hsa04668	TNF signaling pathway	4	7.27E-04
		hsa04650	Natural killer cell mediated cytotoxicity	4	0.001065
Panacis Quinquefolii Radix	Biological Process	GO:0043200	response to amino acid	2	0.005528
		GO:0097192	extrinsic apoptotic signaling pathway in absence of ligand	2	0.006062
		GO:0051092	positive regulation of NF-kappaB transcription factor activi	2	0.023575
	Cellular Component	GO:0045121	membrane raft	2	0.033531
	KEGG pathway	hsa05416	Viral myocarditis	2	0.024656
		hsa04064	NF-kappa B signaling pathway	2	0.037469
		hsa04668	TNF signaling pathway	2	0.045948
Codonopsis Radix	Biological Process	GO:0042493	response to drug	10	4.51E-11
		GO:0018105	peptidyl-serine phosphorylation	8	1.14E-10
		GO:0045893	positive regulation of transcription, DNA-templated	10	4.74E-09
		GO:0051090	regulation of sequence-specific DNA binding transcription factor activity	5	2.25E-08
		GO:0018107	peptidyl-threonine phosphorylation	5	1.30E-07
	Cellular Component	GO:0005654	nucleoplasm	16	1.20E-08
		GO:0005634	nucleus	19	1.58E-07
		GO:0005829	cytosol	15	1.35E-06
		GO:0005667	transcription factor complex	5	6.34E-05
		GO:0043234	protein complex	6	8.70E-05
	Molecular Function	GO:0004693	cyclin-dependent protein serine/threonine kinase activity	6	4.85E-10
		GO:0004674	protein serine/threonine kinase activity	8	2.29E-07
		GO:0004672	protein kinase activity	7	3.67E-06
		GO:0016301	kinase activity	6	9.60E-06
		GO:0005515	protein binding	21	2.23E-05
	KEGG pathway	hsa05161	Hepatitis B	10	8.84E-11
		hsa04660	T cell receptor signaling pathway	8	7.37E-09
		hsa05133	Pertussis	7	4.71E-08
		hsa05203	Viral carcinogenesis	9	5.03E-08
		hsa04914	Progesterone-mediated oocyte maturation	7	1.16E-07
Pseudostellariae radix	Biological Process	GO:0042493	response to drug	9	2.22E-10
		GO:0045893	positive regulation of transcription, DNA-templated	7	7.50E-06
		GO:0032496	response to lipopolysaccharide	5	1.89E-05
		GO:0045944	positive regulation of transcription from RNA polymerase II promoter	7	2.77E-04
		GO:0006954	inflammatory response	5	4.82E-04
	Cellular Component	GO:0005667	transcription factor complex	4	7.13E-04
		GO:0005654	nucleoplasm	9	0.001951
		GO:0005634	nucleus	12	0.002961
		GO:0005829	cytosol	8	0.023221
		GO:0005615	extracellular space	5	0.032528
	Molecular Function	GO:0019899	enzyme binding	6	1.48E-05
		GO:0070412	R-SMAD binding	3	1.98E-04
		GO:0005515	protein binding	17	2.50E-04
		GO:0042802	identical protein binding	6	6.72E-04
		GO:0003677	DNA binding	6	0.021361
	KEGG pathway	hsa05161	Hepatitis B	7	5.32E-07
		hsa05169	Epstein-Barr virus infection	6	6.04E-06
		hsa04668	TNF signaling pathway	5	8.72E-05
		hsa05200	Pathways in cancer	7	1.64E-04
		hsa04380	Osteoclast differentiation	5	1.91E-04
Astragali Radix	Biological Process	GO:0043066	negative regulation of apoptotic process	12	5.79E-10
		GO:0046777	protein autophosphorylation	9	1.15E-09
		GO:0008283	cell proliferation	10	2.45E-08
		GO:0042493	response to drug	9	9.86E-08
		GO:0006468	protein phosphorylation	10	1.60E-07
	Cellular Component	GO:0005654	nucleoplasm	17	1.18E-05
		GO:0005829	cytosol	18	2.47E-05
		GO:0005634	nucleus	23	2.53E-05
		GO:0030496	midbody	5	9.43E-05
		GO:0043234	protein complex	6	9.34E-04
	Molecular Function	GO:0005524	ATP binding	18	6.29E-10
		GO:0004674	protein serine/threonine kinase activity	9	6.15E-07
		GO:0004672	protein kinase activity	8	6.09E-06
		GO:0005515	protein binding	31	8.61E-06
		GO:0019899	enzyme binding	7	4.75E-05
	KEGG pathway	hsa05200	Pathways in cancer	11	1.71E-06
		hsa04914	Progesterone-mediated oocyte maturation	6	2.25E-05
		hsa05145	Toxoplasmosis	6	7.00E-05
		hsa04114	Oocyte meiosis	6	7.30E-05
		hsa05210	Colorectal cancer	5	1.04E-04
Dioscoreae Rhizoma	Biological Process	GO:0032800	receptor biosynthetic process	2	1.79E-04
		GO:0002740	negative regulation of cytokine secretion involved in immune response	2	3.57E-04
		GO:0034116	positive regulation of heterotypic cell-cell adhesion	2	6.55E-04
		GO:0044130	negative regulation of growth of symbiont in host	2	9.53E-04
		GO:0050715	positive regulation of cytokine secretion	2	0.001489
	Molecular Function	GO:0005125	cytokine activity	2	0.010426
	KEGG pathway	hsa05310	Asthma	2	0.004361
		hsa05143	African trypanosomiasis	2	0.004797
		hsa05330	Allograft rejection	2	0.005379
		hsa05144	Malaria	2	0.007123
		hsa05321	Inflammatory bowel disease	2	0.009304
Glycyrrhizae Radix et Rhizoma	Biological Process	GO:0043066	negative regulation of apoptotic process	12	8.36E-10
		GO:0046777	protein autophosphorylation	9	1.49E-09
		GO:0006468	protein phosphorylation	11	1.45E-08
		GO:0042493	response to drug	9	1.27E-07
		GO:0046677	response to antibiotic	5	4.84E-07
	Cellular Component	GO:0030496	midbody	5	1.06E-04
		GO:0005654	nucleoplasm	15	3.51E-04
		GO:0005829	cytosol	16	6.24E-04
		GO:0005634	nucleus	21	6.56E-04
		GO:0043234	protein complex	5	0.007748
	Molecular Function	GO:0005524	ATP binding	18	1.12E-09
		GO:0004672	protein kinase activity	9	5.52E-07
		GO:0004674	protein serine/threonine kinase activity	9	7.82E-07
		GO:0005515	protein binding	31	3.01E-05
		GO:0016301	kinase activity	6	1.30E-04
	KEGG pathway	hsa05200	Pathways in cancer	10	2.13E-05
		hsa04914	Progesterone-mediated oocyte maturation	6	2.69E-05
		hsa05142	Chagas disease (American trypanosomiasis)	6	6.38E-05
		hsa04668	TNF signaling pathway	6	7.31E-05
		hsa05145	Toxoplasmosis	6	8.35E-05

If there were more than five terms enriched in this category, top five terms were selected according to P value and gene count≧ 5;

Count: the number of enriched genes in each term.

### Construction of PPI Networks and Screening of Hub Genes

Using the STRING online database and Cytoscape software, interacting targets between Ginseng Radix et Rhizoma and DLBCL were filtered into the PPI network complex, containing 14 nodes and 38 edges. Among the 14 nodes, 11 nodes were considered significant, with a combined score ≧ 0.7. CASP3 was identified as the hub gene with the filtering of degree ≧ 5. Similarly, 20 nodes were considered significant among 22 interacting targets between Codonopsis Radix and DLBCL. 14 genes were identified as hub genes with the filtering of degree ≥ 5 criteria, including JUN, CASP3, CDK2, AKT1, MAPK3, MAPK1, CDK5, CCNB1, CDK1, CDK4, CCNA2, FOS, TNF, APP.19 nodes were considered significant among 22 interacting targets between Pseudostellariae Radix and DLBCL. 10 hub genes were identified with the filtering of degree ≥ 5 criteria, including JUN, CASP3, AKT1, CDK5, CDK2, CCNB1, CCNA2, STAT1, VEGFA, CDK1. Besides, 31 nodes were considered significant among 35 interacting targets between Astragali Radix and DLBCL. 14 hub genes were identified with the filtering of degree ≥ 5 criteria, including CASP3, MAPK3, CDK1, AKT1, BIRC5, TOP2A, AURKB, MCL1, PLK1, NEK2, AR, CASP9, CASP8, TYMS. Furthermore, 32 nodes were considered significant among 36 interacting targets between Glycyrrhizae Radix et Rhizoma and DLBCL. 13 genes were identified as hub genes with the filtering of degree ≥ 5 criteria, including CASP3, MAPK3, AKT1, BIRC5, CDK1, TOP2A, MCL1, AURKB, NEK2, IL2, CASP9, BDNF, PLK1. No hub genes were found in Panacis Quinquefolii Radix and Dioscoreae Rhizoma.

### Gene Ontology and Pathway Functional Enrichment Analyses of Hub Genes

**FIGURE 3:**
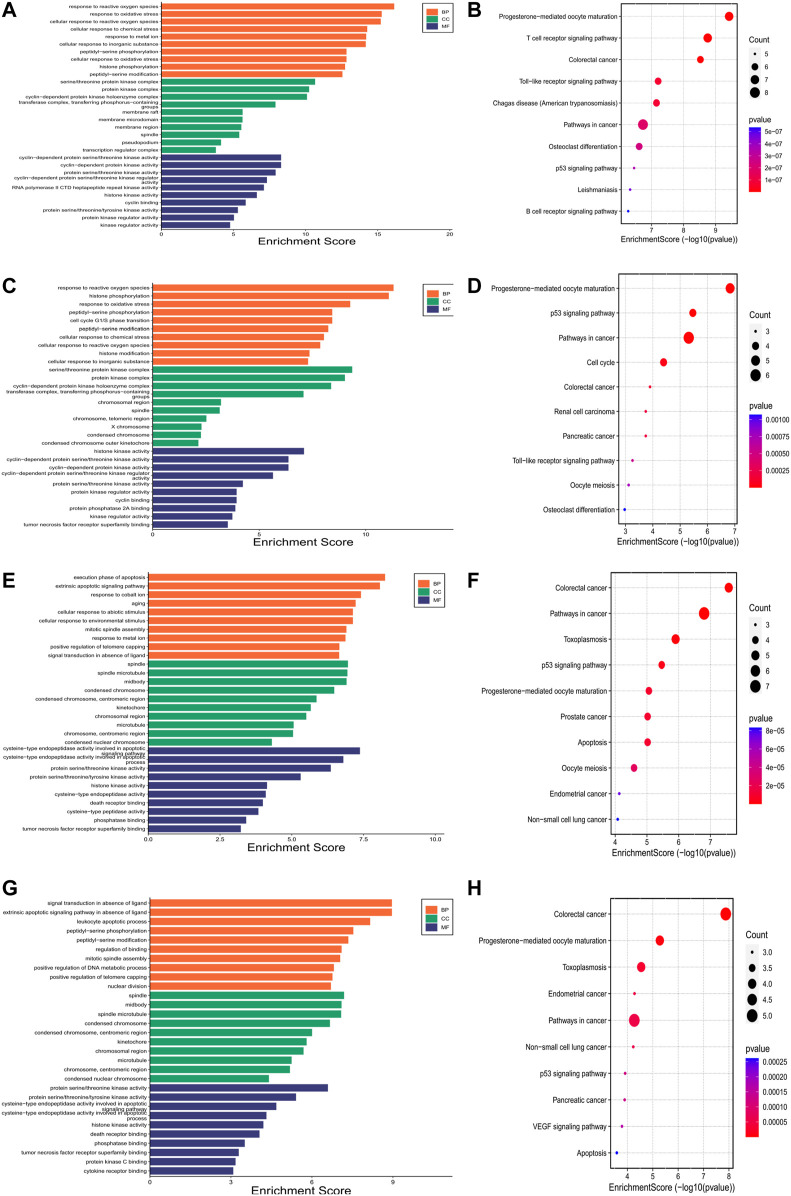
Gene Ontology and Kyoto Encyclopedia of Genes and Genomes analyses of hub genes. Each figure contains top ten pathways of Gene Ontology or Kyoto Encyclopedia of Genes and Genomes. In Gene Ontology enrichment analysis, *Y*-axis is biological process (BP), cellular component (CC) and molecular function (MF), *X*-axis is enrichment score. In Kyoto Encyclopedia of Genes and Genomes analysis, *Y*-axis is pathway name, *X*-axis is enrichment score. Bubble size represents gene numbers involved in the pathway enrichment. Color represents *p*-value, the redder the color, the smaller the *p*-value. **(A)**. Gene Ontology analysis of hub genes for Codonopsis Radix; **(B)**. Pathway enrichment analysis of hub genes for Codonopsis Radix; **(C)**. Gene Ontology analysis of hub genes for Pseudostellariae Radix; **(D)**. Pathway enrichment analysis of hub genes for Pseudostellariae Radix; **(E)**. Gene Ontology analysis of hub genes for Astragali Radix; **(F)**. Pathway enrichment analysis of hub genes for Astragali Radix; **(G)**. Gene Ontology analysis of hub genes for Glycyrrhizae Radix et Rhizoma; **(H)**. Pathway enrichment analysis of hub genes for Glycyrrhizae Radix et Rhizoma.

We also performed GO and KEGG pathway enrichment analyses on the hub genes. Bar charts of GO (BP, CC, MF) and bubble charts of KEGG pathways were obtained. As shown in Figure 3, GO and KEGG enrichment analyses predicted hub genes in Codonopsis Radix mainly involved in response to reactive oxygen species, serine/threonine protein kinase complex, cyclin-dependent protein serine/threonine kinase activity, progesterone-mediated oocyte maturation and so forth; hub genes in Pseudostellariae Radix mainly involved in response to reactive oxygen species, serine/threonine protein kinase complex, histone kinase activity, progesterone-mediated oocyte maturation and so forth; hub genes in Astragali Radix mainly involved in execution phase of apoptosis, spindle, cysteine-type endopeptidase activity involved in apoptotic signaling pathway, colorectal cancer and so forth; while hub genes in Glycyrrhizae Radix et Rhizoma mainly involved in signal transduction in absence of ligand, spindle, protein serine/threonine kinase activity, protein serine/threonine kinase complex, colorectal cancer and so forth. It is shown that these hub genes mainly enriched in cancer-related pathways.

From the top ten KEGG pathway results, the significantly enriched hub genes were CASP3, CDK2, AKT1, MAPK3, MAPK1, CCNB1, CDK1, CCNA2, JUN, CDK4, FOS, TNF in Codonopsis Radix; CASP3, AKT1, CDK2, CCNB1, CCNA2, CDK1, JUN, STAT1, VEGFA in Pseudostellariae Radix; CASP3, MAPK3, AKT1, BIRC5, CASP9, AR, CASP8, PLK1, CDK1 in Astragali Radix; CASP3, MAPK3, AKT1, BIRC5, CASP9, CDK1, PLK1 in Glycyrrhizae Radix et Rhizoma.

### Molecular Docking Verification

**FIGURE 4:**
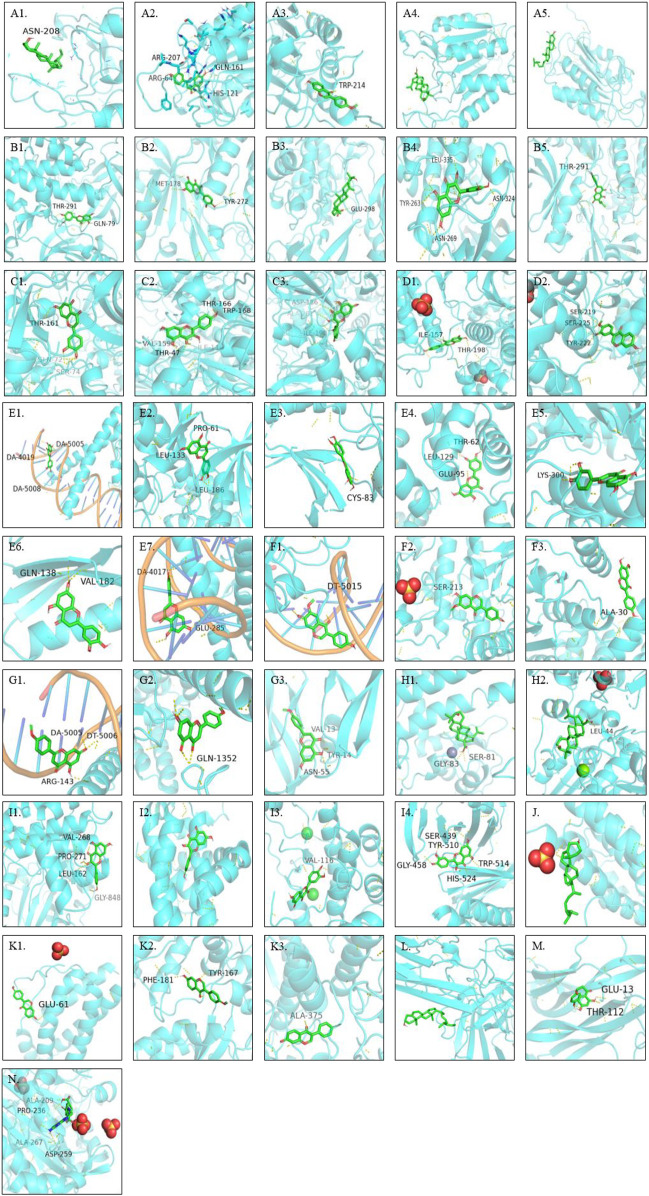
Molecular docking results: A1-A5. CASP3 and beta-sitosterol, luteolin, formononetin, mairin, sitosterol, respectively; B1-B5. AKT1 and luteolin, isorhamnetin, mairin, quercetin,2-(3,4-dihydroxyphenyl)-5,7-dihydroxy-6-(3-methylbut-2-enyl)chromone, respectively; C1-C3. CDK1 and luteolin, kaempferol, quercetin, respectively; D1-D2. MAPK3 and glycitein, calycosin, respectively; E1-E7. luteolin and JUN, CDK2, CDK5, CCNB1, CCNA2, APP, FOS, respectively; F1-F3. glycitein and JUN, MAPK1, CDK4, respectively; G1-G3. acacetin and JUN, STAT1, VEGFA, respectively; H1-H2. mairin and BIRC5, TOP2A, respectively; I1-I4. quercetin and AUKRB, MCL1, NEK2, PLK1, respectively; J. hederagenin and AR; K1-K3. formononetin and IL2, CASP8, CASP9, respectively; L. stigmasterol and TNF; M. naringenin and BDNF; N. FA and TYMS.

Through AutoDock Vina, all the hub genes and their related active ingredients were run for molecular docking. As shown in Figure 4, 16 active ingredients and 28 hub genes were run via AutoDock Vina for molecular docking. The spatial coordinates and the lowest binding affinity are shown in Table 3. Results in Figure 4 showed that in Ginseng Radix et Rhizoma, beta-sitosterol had a good affinity to CASP3 through hydrogen bonding. In Codonopsis Radix, luteolin might suppress cancer cell growth through hydrogen-bonding with AKT1, APP, CASP3, CCNA2, CCNB1, CDK1, CDK2, CDK5, FOS, JUN; glycitein could hydrogen-bond with CDK4, JUN, MAPK1, MAPK3; while stigmasterol only bound with TNF by van der Waals forces. In Pseudostellariae Radix, good affinities were also detected between luteolin and hub genes, including AKT1, CASP3, CCNA2, CCNB1, CDK1, CDK2, CDK5, JUN. Besides, acacetin and JUN, STAT1, VEGFA were bound together by hydrogen bonds. Beta-sitosterol took effect through CASP3. In Astragali Radix, quercetin might hold together with AKT1, AUKRB, CDK1, NEK2, PLK1 through hydrogen bonds, with MCL1 by van der Waals forces; mairin was bound with AKT1, BIRC5, TOP2A by hydrogen bonds, with CASP3 by van der Waals forces. Formononetin might induce cancer apoptosis through hydrogen-bonding with CASP3, CASP8, CASP9. Isorhamnetin, kaempferol, FA, calycosin, hederagenin might play an anti-tumor role through targeting AKT1, CDK1 TYMS, MAPK3, AR, respectively. As for Glycyrrhizae Radix et Rhizoma, similar results were found in quercetin, formononetin, isorhamnetin, kaempferol, calycosin and mairin. Meanwhile, CASP3, AKT1, IL2, BDNF could also bond with sitosterol, formononetin, naringenin, 2-(3,4-dihydroxyphenyl)-5,7-dihydroxy-6-(3-methylbut-2-enyl) chromone, respectively.

**TABLE 3:** Docking results of active ingredients with target proteins.

Targets	PDB-ID	Ligands	Affinity (kcal/mol)	Center coordinates(x,y,z)/nm
CASP3	3KJF	beta-sitosterol	−5.0	10.319 6.913 2.268
CASP3	3KJF	luteolin	−7.5	10.319 6.913 2.268
CASP3	3KJF	formononetin	−7.3	10.319 6.913 2.268
CASP3	3KJF	mairin	−8.2	10.319 6.913 2.268
CASP3	3KJF	sitosterol	−3.5	10.319 6.913 2.268
AKT1	6S9W	luteolin	−10.1	−13.631 −12.55 15.018
AKT1	6S9W	isorhamnetin	−9.9	−13.631 −12.55 15.018
AKT1	6S9W	mairin	−7.6	−13.631 −12.55 15.018
AKT1	6S9W	quercetin	−7.7	−13.631 −12.55 15.018
AKT1	6S9W	2-(3,4-dihydroxyphenyl)-5,7-dihydroxy-6-(3-methylbut-2-enyl)chromone	−10.6	−13.631 −12.55 15.018
CDK1	4YC6	luteolin	−8.4	−14.325 12.348 −22.28
CDK1	4YC6	kaempferol	−7.7	−14.325 12.348 −22.28
CDK1	4YC6	quercetin	−7.9	−14.325 12.348 −22.28
MAPK3	6GES	glycitein	−8.1	46.838 4.48 −8.117
MAPK3	6GES	calycosin	−8.1	46.838 4.48 −8.117
JUN	1A02	luteolin	−8.4	28.223 28.762 60.016
CDK2	6GUE	luteolin	−9.6	−29.87 −7.262 18.182
CDK5	3O0G	luteolin	−9.3	16.519 34.169 52.349
CCNB1	2B9R	luteolin	−7.0	−65.873 41.523 −11.34
CCNA2	5IF1	luteolin	−7.8	−61.902 -39.304 −11.927
APP	1OWT	luteolin	−6.3	−0.013 -0.096 0.004
FOS	1A02	luteolin	−8.4	28.223 28.762 60.016
JUN	1A02	glycitein	−7.4	28.223 28.762 60.016
MAPK1	1PME	glycitein	−7.1	−4.477 8.779 47.428
CDK4	4YC6	glycitein	−8.4	6.503 5.666 29.291
JUN	1A02	acacetin	−7.9	28.223 28.762 60.016
VEGFA	3QTK	acacetin	−7.8	31.497 18.909 7.02
STAT1	1YVL	acacetin	−8.0	−15.938 −29.561 171.162
BIRC5	2RAX	mairin	−7.1	26.692 −61.209 4.957
TOP2A	1ZXN	mairin	−7.5	41.327 23.743 40.984
AUKRB	4AF3	quercetin	−6.1	15.553 −16.602 −3.528
MCL1	6B4U	quercetin	−7.6	−8.597 15.908 10.321
NEK2	2XKD	quercetin	−6.9	15.251 −11.912 17.204
PLK1	2OGQ	quercetin	−7.1	55.303 16.8 27.793
AR	2PIV	hederagenin	−5.3	21.023 5.462 11.667
IL2	1M47	formononetin	−5.6	6.751 25.891 14.188
CASP8	5JQE	formononetin	−7.0	169.678 29.358 -0.457
CASP9	2AR9	formononetin	−8.5	19.147 38.329 27.142
TNF	3WD5	stigmasterol	−5.8	−20.065 21.641 22.187
BDNF	1B8M	naringenin	−6.8	2.715 23.339 14.231
TYMS	3N5E	FA	−7.1	−48.904 0.049 11.859

### Correlation Between Hub Genes and Survival Time

**FIGURE 5:**
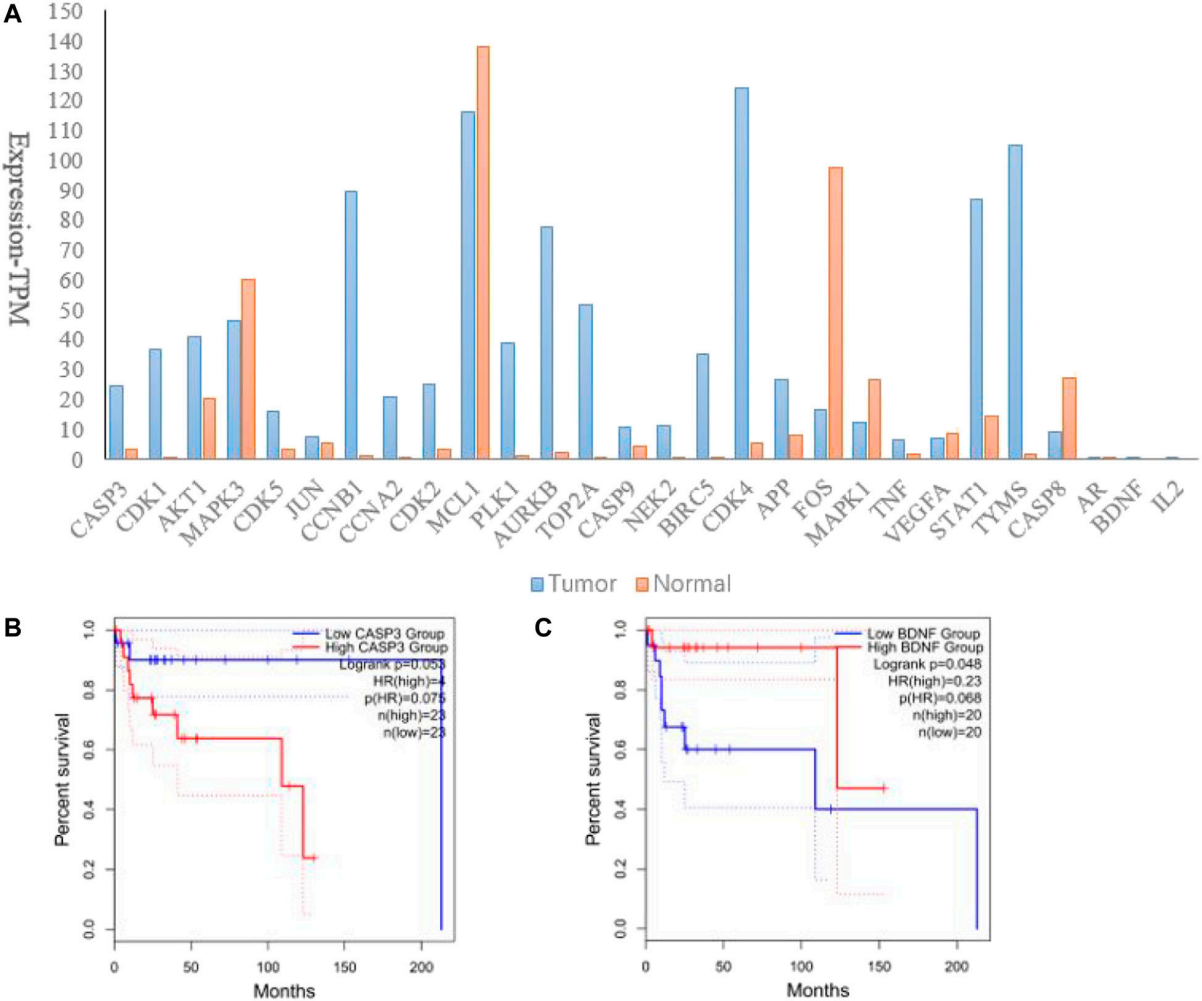
Verification of hub genes in the GEPIA 2.0 database. **(A)**. Expression of hub genes in DLBCL as compared to those in non-tumor tissues; **(B)**. Correlation between CASP3 and disease-free survival in DLBCL; **(C)**. Correlation between BDNF and disease-free survival in DLBCL.

In order to discover whether hub genes had correlations with DLBCL prognosis, hub genes were input into the GEPIA 2.0 database. Firstly, we tested their transcription levels in datasets concerning DLBCL vs non-tumor tissues. As shown in Figure 5A, all the hub genes were differently expressed in DLBCL as compared to those in non-tumor tissues. Then comparison of each gene’s survival curves was made and *p* values were calculated by log-rank. Results showed that only alteration in CASP3 and BDNF correlated with disease-free survival, for their *p*-values of log-rank were less than or close to 0.05, when group cutoff was set to 50% (Figures 5B,C). In view of the small number of samples, a retrospective study with larger sample is expected to confirm the conclusion.

### Common Target Genes and Active Ingredients of Different Qi-Invigorating Herbs

**FIGURE 6:**
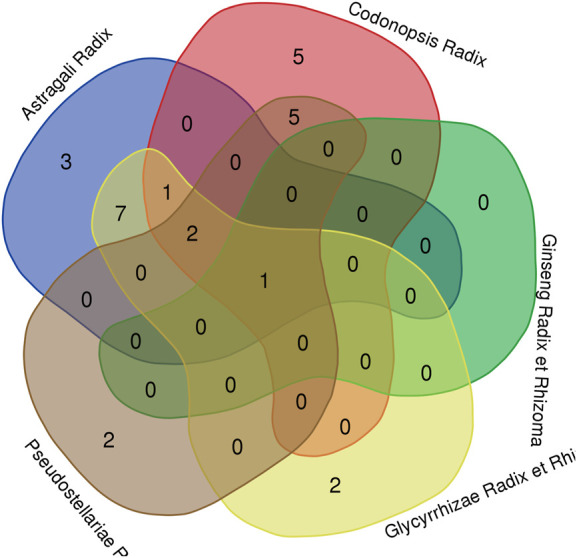
A Venn diagram for common hub genes. The red part represents Codonopsis Radix; the green part represents Ginseng Radix et Rhizoma; the yellow part represents Glycyrrhizae Radix et Rhizoma; the brown part represents Pseudostellariae Radix; the blue part represents Astragali Radix.

It is noteworthy that different Qi-invigorating herbs exist same active ingredients, i.e. different Qi-invigorating herbs may target same genes in DLBCL. Thus, we first took the intersection of hub gene related ingredients. However, no common hub gene related ingredients were found. Subsequently, additional analysis was performed on hub genes. Through R software 4.1.0, it was detected that four hub genes (CASP3, CDK1, AKT1, MAPK3) corresponded to at least three of five Qi-invigorating herbs (Figure 6). Therefore, it is suggested that CASP3, CDK1, AKT1 and MAPK3 may be common target genes for different Qi-invigorating herbs acting on DLBCL.

### Experimental Verification

#### Cytotoxicity Study of Qi-Invigorating Herbs in Diffuse Large B Cell Lymphoma Cells

In order to verify the effect of common hub genes in Qi-invigorating herbs, an *in vitro* experiment was carried out. Firstly, we evaluated the cytotoxic efficacy of Qi-invigorating herbs in two DLBCL cell lines at concentrations between 3 and 10 mg/ml. Results showed that the IC50 values were 3.91 mg/ml, 4.01 mg/ml for Astragali Radix; 2.4 mg/ml, 1.94 mg/ml for Glycyrrhizae Radix et Rhizoma; 2.05 mg/ml, 3.2 mg/ml for Ginseng Radix et Rhizoma; 1.73 mg/ml, 4.11 mg/ml for Pseudostellariae Radix; 3.23 mg/ml, 4.1 mg/ml for Codonopsis Radix in SUDHL2 and SUDHL6 cells, respectively. Considering the cytotoxicity of all Qi-invigorating herbs, 5 mg/ml concentration of Qi-invigorating herbs was selected as a suitable intervention concentration for subsequent experiments.

#### The Role of Qi-Invigorating Herbs in the Proliferation of Diffuse Large B Cell Lymphoma Cells

**FIGURE 7:**
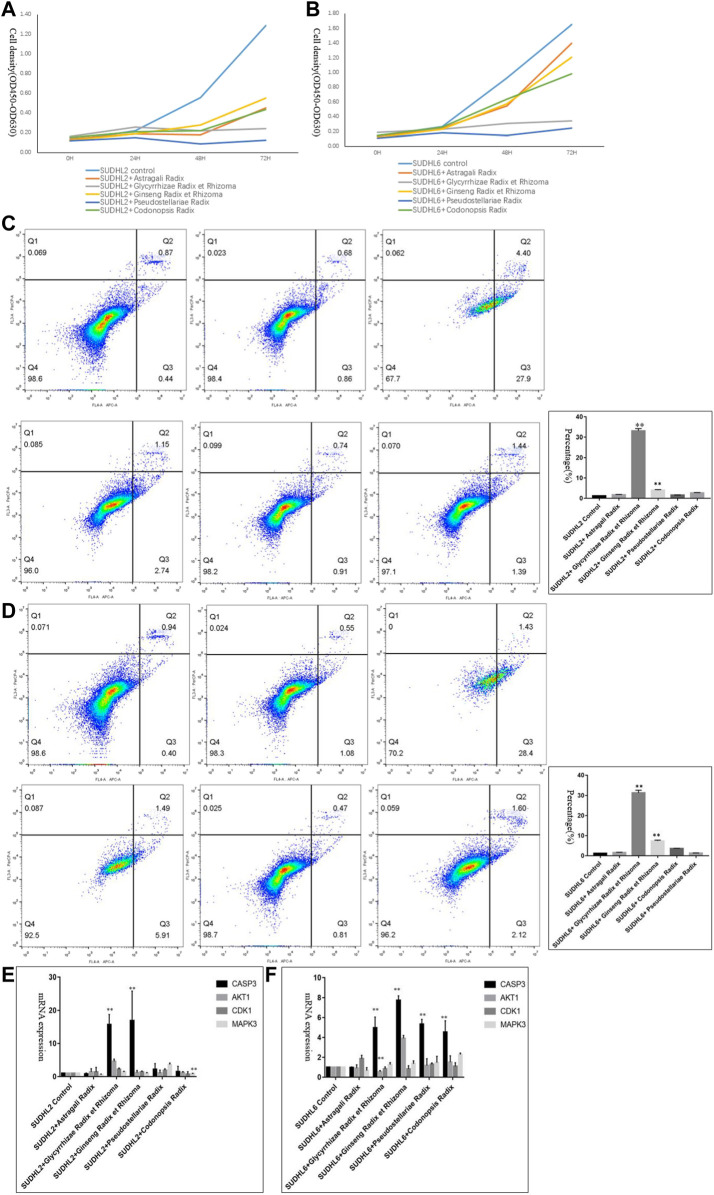
Experimental validation *in vitro*. **(A)**. The proliferative ability of SUDHL2 cells incubating with different Qi-invigorating herbs; **(B)**. The proliferative ability of SUDHL6 cells incubating with different Qi-invigorating herbs; **(C)**. The cell apoptosis of SU-DHL-2 cells incubating with different Qi-invigorating herbs was measured through flow cytometry analysis (From upper left to right: Negative control, SUDHL2 cells treated with Astragali Radix, SUDHL2 cells treated with Glycyrrhizae Radix et Rhizoma; From lower left to right: SUDHL2 cells treated with Ginseng Radix et Rhizoma, SUDHL2 cells treated with Pseudostellariae Radix, SUDHL2 cells treated with Codonopsis Radix); **(D)**. The cell apoptosis of SU-DHL-6 cells incubating with different Qi-invigorating herbs was measured through flow cytometry analysis (From upper left to right: Negative control, SUDHL6 cells treated with Astragali Radix, SUDHL6 cells treated with Glycyrrhizae Radix et Rhizoma; from lower left to right: SUDHL6 cells treated with Ginseng Radix et Rhizoma, SUDHL6 cells treated with Pseudostellariae Radix, SUDHL6 cells treated with Codonopsis Radix); **(E)**. mRNA expression of CASP3, AKT1, CDK1, MAPK3 in SUDHL2 cells incubating with different Qi-invigorating herbs; **(F)**. mRNA expression of CASP3, AKT1, CDK1, MAPK3 in SUDHL6 cells incubating with different Qi-invigorating herbs. *p < 0.05 and **p < 0.01.

CCK-8 assays were applied to determine the effects of Astragali Radix, Glycyrrhizae Radix et Rhizoma, Ginseng Radix et Rhizoma, Pseudostellariae Radix and Codonopsis Radix on DLBCL cell proliferation. Results showed that the proliferation ability of SU-DHL-2 and SU-DHL-6 cells was decreased via incubating with Qi-invigorating herbs (Figures 7A,B).

#### The Role of Qi-Invigorating Herbs in the Apoptosis of Diffuse Large B Cell Lymphoma Cells

To measure the induction of apoptosis, cells were treated with 5 mg/ml of Astragali Radix, Glycyrrhizae Radix et Rhizoma, Ginseng Radix et Rhizoma, Pseudostellariae Radix and Codonopsis Radix decoctions for 48 h, respectively. Flow cytometry analysis exhibited that incubation with Glycyrrhizae Radix et Rhizoma resulted in a remarkable rise in cell apoptosis [(33.09 ± 0.99)% in SUDHL2 and (31.3 ± 1.3)% in SUDHL6], comparing to negative control groups (Figures 7C,D). Meanwhile cell apoptosis in DLBCL cells treated with Ginseng Radix et Rhizoma was slightly elevated (Figures 7C,D). Thus it is suggested that some Qi-invigorating herbs participated in the apoptosis of DLBCL cells.

#### The Role of Common Hub Genes in Diffuse Large B Cell Lymphoma Cells Treated With Qi-Invigorating Herbs

In order to further elaborate the roles of common hub genes, we tested CASP3, CDK1, AKT1 and MAPK3 expression in DLBCL cells incubated with Qi-invigorating herbs, respectively. As shown in Figures 7E,F, DLBCL cells treated with Glycyrrhizae Radix et Rhizoma or Ginseng Radix et Rhizoma had an effectively increase in CASP3 at mRNA levels compared with negative control groups, suggesting that Qi-invigorating herbs regulated DLBCL cell apoptosis mainly through targeting CASP3. It was noteworthy that most CDK1, AKT1, MAPK3 expression did not change significantly in DLBCL cells treated with Qi-invigorating herbs (Figures 7E,F). Therefore, it is concluded that CDK1, AKT1 and MAPK3 are not common hub genes in DLBCL cells treated with Qi-invigorating herbs.

### Tumor Microenvironment Analysis

**FIGURE 8:**
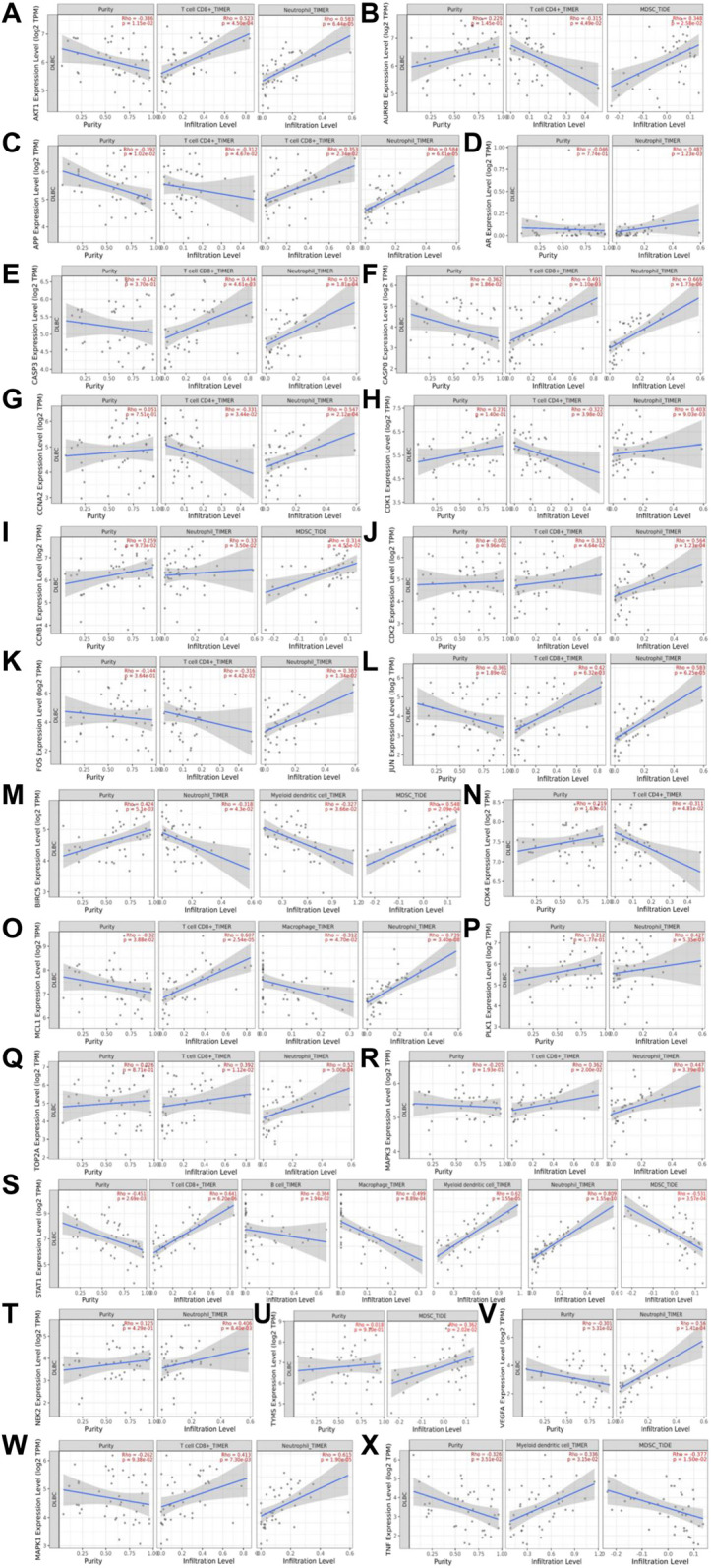
The relationship between hub genes and tumor-infiltrating immune cells. **(A)**. The relationship between AKT1 and tumor-infiltrating immune cells (CD8^+^ T cells and neutrophils); **(B)**. The relationship between AURKB and tumor-infiltrating immune cells (CD4^+^ T cells and MDSCs); **(C)**. The relationship between APP and tumor-infiltrating immune cells (CD4^+^ T cells, CD8^+^ T cells and neutrophils); **(D)**. The relationship between AR and neutrophils; **(E)**. The relationship between CASP3 and tumor-infiltrating immune cells (CD8^+^ T cells and neutrophils); **(F)**. The relationship between CASP8 and tumor-infiltrating immune cells (CD8^+^ T cells and neutrophils); **(G)**. The relationship between CCNA2 and tumor-infiltrating immune cells (CD4^+^ T cells and neutrophils); **(H)**. The relationship between CDK1 and tumor-infiltrating immune cells (CD4^+^ T cells and neutrophils); **(I)**. The relationship between CCNB1 and tumor-infiltrating immune cells (MDSCs and neutrophils); **(J)**. The relationship between CDK2 and tumor-infiltrating immune cells (CD8^+^ T cells and neutrophils); **(K)**. The relationship between FOS and tumor-infiltrating immune cells (CD4^+^ T cells and neutrophils); **(L)**. The relationship between JUN and tumor-infiltrating immune cells (CD8^+^ T cells and neutrophils); **(M)**. The relationship between BIRC5 and tumor-infiltrating immune cells (neutrophils, myeloid dendritic cells and MDSCs); **(N)**. The relationship between CDK4 and CD4^+^ T cells; **(O)**. The relationship between MCL1 and neutrophils, CD8^+^ T cells, macrophage; **(P)**. The relationship between PLK1 and neutrophils; **(Q)**. TOP2A and tumor-infiltrating immune cells (CD8^+^ T cells and neutrophils); **(R)**. The relationship between MAPK3 and tumor-infiltrating immune cells (CD8^+^ T cells and neutrophils); **(S)**. The relationship between STAT1 and tumor-infiltrating immune cells (B cells, CD8^+^ T cells, macrophage, myeloid dendritic cells, neutrophils, MDSCs); **(T)**. The relationship between NEK2 and neutrophils; **(U)**. The relationship between TYMS and MDSCs; **(V)**. The relationship between VEGFA and neutrophils; **(W)**. MAPK1 and tumor-infiltrating immune cells (CD8^+^ T cells and neutrophils); **(X)**. The relationship between TNF and myeloid dendritic cells, MDSCs. Myeloid derived suppressor cells: MDSC.

To further explore the mechanisms of Qi-invigorating herbs acting on DLBCL TME, we conducted the analysis of the relationship between hub genes and tumor-infiltrating immune cells (B cells, CD4^+^ T cells, CD8^+^ T cells, neutrophils, macrophages, myeloid DCs, MDSCs) from the TIMER 2.0 database. As shown in Figure 8, the expression of AKT1 was negatively associated with tumor purity (*r* = −0.386, *p* = 1.15e-02), positively correlated with CD8^+^ T cells (*r* = 0.523, *p* = 4.50e-04) and neutrophils (*r* = 0.583, *p* = 6.44e-05) in infiltrating levels. Similar relationships were found in CASP8, JUN, APP and MCL1 (*p* < 0.05). CASP3, CDK2, MAPK1, TOP2A, MAPK3 only had positive significant correlation with CD8^+^ T cells and neutrophils (*r* > 0, *p* < 0.01). CDK1, CCNA2, FOS had statistical correlation with CD4^+^ T cells and neutrophils (*p* < 0.05), while BIRC5 and CCNB1 correlated with both neutrophils and MDSCs (*p* < 0.05). PLK1, VEGFA, AR only correlated with neutrophils (*p* < 0.05), TYMS, AURKB, TNF mainly correlated with MDSCs (*p* < 0.05). The expression of STAT1 was negatively associated with tumor purity (*r* = −0.451, *p* = 2.69e-03), B cells (*r* = −0.364, *p* = 1.94e-02), macrophage (*r* = −0.499, *p* = 8.89e-04) and MDSCs (*r* = −0.531, *p* = 3.57e-04) in infiltrating levels, but positively correlated with CD8^+^ T cells (*r* = 0.641, *p* = 6.20e-06), myeloid dendritic cells (*r* = 0.62, *p* = 1.55e-05) and neutrophils (*r* = 0.809, *p* = 1.55e-10) in infiltrating levels. However, no correlation was found among BDNF, CDK5, IL2 and tumor-infiltrating immune cells in DLBCL. Since most hub genes were associated with neutrophils in infiltrating levels, we speculated that Qi-invigorating herbs mainly affects DLBCL microenvironment through acting on neutrophils.

## Discussion

TCM is a very important cancer treatment strategy in China. It has been widely reported that TCM herbs can improve DLBCL survival and provide a complementary therapy for cases that are refractory or have relapsed. According to the TCM theory, disturbance of Yin-Yang is one of the main reasons for carcinogenesis. To alleviate symptoms of disease, TCM aims to restore the balance of Yin and Yang in addition to eliminate pathogenic factors (“Quxie”), which is somewhat similar to TME from the view of theory. Based on preclinical studies, Qi-invigorating herbs have been proved to have immune enhancement and multi-target regulation. Therefore, a systematic study of Qi-invigorating herbs in DLBCL would elucidate their mechanisms and provide guidance for herb selection, or even personalized therapy.

In this study, eight commonly used Qi-invigorating herbs were included, which have been widely employed in herbal prescriptions for cancer treatment. Active ingredients were first selected from the TCMSP database. Then potential targets were output via the SwissTargetPrediction and STITCH databases. By intersection with target genes of DLBCL, it was found that besides Atractylodis Macrocephalae Rhizoma, the other seven Qi-invigorating herbs could act on DLBCL. GO and KEGG pathway enrichment results revealed that there were some similarities as well as differences among Qi-invigorating herbs.

Through PPI construction networks, hub genes were output, which implicated Ginseng Radix et Rhizoma, Codonopsis Radix, Pseudostellariae Radix, Astragali Radix, Glycyrrhizae Radix et Rhizoma might be more appropriate for herbal prescriptions in treating DLBCL. The suppressed proliferation by these Qi-invigorating herbs *in vitro* verified this view. Excellent molecular docking results for hub genes and their corresponding ingredients further confirmed this view.

Interestingly, CASP3, CDK1, AKT1 and MAPK3 were considered as common target genes. Among them, CASP3 is one important member of caspases, characterized by programmed cell death (Galluzzi et al., 2016). It is often used as a marker for efficacy of cancer therapy. In TME, it has been indicated that CASP3 activation could trigger pyroptosis, a form of cell death that is critical for immunity by cleaving gasdermin E (GSDME) in GSDME-expressing tumors (Wang et al., 2017; Zhang et al., 2020). Similar finding was observed in T-cell lymphoma, where immunogenic cell death depended on CASP3 activity, with reduced antitumor immunity generated by CASP3-deficient EL4 cells (Jaime-Sanchez et al., 2020). Our study detected that DLBCL cells treated with Glycyrrhizae Radix et Rhizoma or Ginseng Radix et Rhizoma promoted their apoptosis accompanied by up-regulated CASP3. Thus it is concluded that Qi-invigorating herbs regulated DLBCL cell apoptosis mainly through targeting CASP3. In view of the correlation between CASP3 and DLBCL prognosis, it is of great significance to further explore the mechanisms of Glycyrrhizae Radix et Rhizoma and Ginseng Radix et Rhizoma acing on CASP3 in DLBCL.

Although CDK1, AKT1 and MAPK3 were considered as other common hub genes for Qi-invigorating herbs, most CDK1, AKT1, MAPK3 expression did not change significantly *in vitro*. Therefore, it is concluded that CDK1, AKT1 and MAPK3 are not common hub genes in DLBCL cells treated with Qi-invigorating herbs. In this study we have well described the effect of common hub genes by wet lab experiments. The effects and mechanisms of other hub genes required further verification in the future. The detailed mechanisms obtained by experiments may provide a guidance for herb selection, prescription formation, new drug design, or even personalized therapy.

Molecular docking results revealed that one common hub gene exhibited good binding energy for multiple active ingredients and one ingredient targeted more than one hub gene. Based on binding affinity and specific ligand-receptor residue interactions, there are six active ingredients (luteolin, glycitein, acacetin, quercetin, mairin, formononetin) targeting more than three hub genes in each Qi-invigorating herb, indicating they have potential as DLBCL drugs. So far, some of these ingredients have been well described for their anti-lymphoma effects in experimental studies. luteolin, an active ingredient extracted from Codonopsis Radix and Pseudostellariae Radix, has been tested to inhibit the proliferation of classical Hodgkin’s lymphoma cells via caspase activation (Gharbaran et al., 2020). Quercetin, an active ingredient of Astragali Radix and Glycyrrhizae Radix et Rhizoma, has been detected to inhibit cancer cell growth by modulating AKT and NF-κB pathways in lymphoma models (Soofiyani et al., 2021). The experimental results also displayed the prospect of Qi-invigorating herbs in DLBCL treatment. Thus, more pharmacodynamic studies are required to examine their effects in DLBCL models.

Besides directly targeting cancer cells, previous evidence suggested that Qi-invigorating herbs could exert antitumor effects by upregulating immune responses even in immunosuppressive TME. Therefore, to explore the mechanisms of Qi-invigorating herbs for DLBCL treatment in depth, we conduct a comprehensive TME analysis for hub genes. In DLBCL microenvironment, although much is still unknown concerning the composition of immune cells, CD4^+^ T cells, CD8^+^ T cells, neutrophils, macrophages, myeloid DCs and MDSCs have been widely tested as independent predictors of DLBCL outcome (Azzaoui et al., 2016; Chen et al., 2016; Judd et al., 2017; Kusano et al., 2017; Ciavarella et al., 2018; Staiger et al., 2020; Merdan et al., 2021; Xu et al., 2021). Manfroi et al. (Manfroi et al., 2017) further demonstrated an association between neutrophils and A Proliferation-Inducing TNF Ligand (APRIL). They reported that APRIL produced by DLBCL-related neutrophils increased tumor aggressiveness and affected disease prognosis (Manfroi et al., 2017). In our study, the relationship between hub genes and tumor-infiltrating immune cells (B cells, CD4^+^ T cells, CD8^+^ T cells, neutrophils, macrophages, myeloid DCs and MDSCs) was tested via the TIMER 2.0 database. Since most hub genes were found to be associated with neutrophils in infiltrating levels, it was reasonable to speculate that Qi-invigorating herbs mainly affected DLBCL TME through acting on tumor-associated neutrophils. Further research is needed to validate our findings.

In a word, this study systematically explored the active ingredients, potential targets, hub genes and the impact of immune cells in Qi-invigorating herbs for DLBCL treatment. Hub genes and immune infiltrating cells provided the molecular basis for each Qi-invigorating herb acting on DLBCL. Study on common mechanisms revealed that CASP3 might be the common target of Qi-invigorating herbs on DLBCL apoptosis. Tumor-associated neutrophils may be main target cells of DLBCL treated by Qi-invigorating herbs. Although this study provided preliminary predictions, it was important for the modernization of TCM. Such researches would undoubtedly increase our understanding of DLBCL pathogenesis. However, the conclusions still need to be verified with subsequent wet lab experiments.

## Conclusion

Through network pharmacology analysis and experimental validation, Qi-invigorating herbs were proved to be appropriate for DLBCL treatment. Potential targets, hub genes and targeted immune cells of Qi-invigorating herbs helped us to understand the mechanisms. Study on common mechanisms revealed that CASP3 might be the common target of Qi-invigorating herbs on DLBCL apoptosis. Tumor-associated neutrophils may be main target cells of DLBCL treated by Qi-invigorating herbs. However, the mechanisms of hub genes still required verification, which may provide a guidance for herb selection, prescription formation, new drug design, or even personalized therapy.

In all, our findings may provide a theoretical basis for the application of Qi-invigorating herbs in DLBCL. This article may provide a reference and clinical transformation value for TCM.

## Data Availability

The original contributions presented in the study are included in the article/Supplementary Materials, further inquiries can be directed to the corresponding author.
