# Supplementary material for: Impact of Qi-Invigorating Traditional Chinese Medicines on Diffuse Large B Cell Lymphoma Based on Network Pharmacology and Experimental Validation
Source: Front Pharmacol. 2021 Dec 9;12:787816. doi: 10.3389/fphar.2021.787816 (PMC8699731; doi:10.3389/fphar.2021.787816)
Supplement: Supplementary file 1 [file Table1.docx]

Supplementary Table 1. The details of active ingredients of Qi-invigorating herbs

| Herbs | Compounds | OB | DL | HL |
| --- | --- | --- | --- | --- |
| Ginseng Radix et Rhizoma | stigmasterol | 43.830 | 0.757 | 5.575 |
|  | beta-sitosterol | 36.914 | 0.751 | 5.355 |
|  | Inermin | 65.831 | 0.538 | 11.734 |
|  | kaempferol | 41.882 | 0.241 | 14.743 |
|  | Chrysanthemaxanthin | 38.724 | 0.584 | 17.475 |
|  | Celabenzine | 101.883 | 0.488 | 8.150 |
|  | Deoxyharringtonine | 39.274 | 0.812 | 7.898 |
|  | Dianthramine | 40.446 | 0.197 | 5.144 |
|  | arachidonate | 45.573 | 0.205 | 7.562 |
|  | Frutinone A | 65.904 | 0.342 | 19.101 |
|  | ginsenoside rh2 | 36.320 | 0.559 | 11.076 |
|  | Ginsenoside-Rh4_qt | 31.112 | 0.778 | 6.973 |
|  | Girinimbin | 61.215 | 0.315 | 8.168 |
|  | Gomisin B | 31.990 | 0.829 | 7.806 |
|  | malkangunin | 57.714 | 0.626 | 4.092 |
|  | Panaxadiol | 33.088 | 0.794 | 6.342 |
|  | suchilactone | 57.519 | 0.556 | 9.035 |
|  | alexandrin_qt | 36.914 | 0.753 | 5.531 |
|  | ginsenoside Rg5_qt | 39.563 | 0.785 | 5.654 |
|  | Fumarine | 59.263 | 0.827 | 23.465 |
| Panacis Quinquefolii Radix | (2R,3S,4S,5R,6R)-2-(hydroxymethyl)-6-[[(3S,5R,8R,9R,10R,12R,13R,14R,17S)-12-hydroxy-4,4,8,10,14-pentamethyl-17-[(2S)-6-methyl-2-[(2S,3R,4S,5S,6R)-3,4,5-trihydroxy-6-(hydroxymethyl)oxan-2-yl]oxyhept-5-en-2-yl]-2,3,5,6,7,9,11,12,13,15,16,17-dodecahydro-1H-c | 36.432 | 0.253 | 12.459 |
|  | PQ-2 | 36.740 | 0.194 | 4.263 |
|  | (8S,9S,10R,13R,14S,17R)-17-[(1R,4R)-4-ethyl-1,5-dimethylhexyl]-10,13-dimethyl-1,2,8,9,11,12,14,15,16,17-decahydrocyclopenta[a]phenanthren-7-one | 43.867 | 0.746 | 4.838 |
|  | beta-sitosterol | 36.914 | 0.751 | 5.355 |
|  | ginsenoside rh2 | 36.320 | 0.559 | 11.076 |
|  | stigmast-7-enol | 37.423 | 0.751 | 6.284 |
|  | papaverine | 64.043 | 0.378 | 4.143 |
|  | daucosterol_qt | 36.914 | 0.753 | 5.598 |
|  | Daturilin | 50.365 | 0.768 | 5.732 |
| Codonopsis Radix | poriferasta-7,22E-dien-3beta-ol | 42.979 | 0.756 | 5.485 |
|  | Perlolyrine | 65.948 | 0.275 | 12.618 |
|  | ZINC03978781 | 43.830 | 0.756 | 5.789 |
|  | Stigmasterol | 43.830 | 0.757 | 5.575 |
|  | 7-Methoxy-2-methyl isoflavone | 42.565 | 0.199 | 16.887 |
|  | Spinasterol | 42.979 | 0.755 | 5.321 |
|  | Chrysanthemaxanthin | 38.724 | 0.584 | 17.475 |
|  | Frutinone A | 65.904 | 0.342 | 19.101 |
|  | luteolin | 36.163 | 0.246 | 15.944 |
|  | stigmast-7-enol | 37.423 | 0.751 | 6.284 |
|  | 3-beta-Hydroxymethyllenetanshiquinone | 32.161 | 0.409 | 22.509 |
|  | methyl icosa-11,14-dienoate | 39.667 | 0.229 | 5.237 |
|  | 5alpha-Stigmastan-3,6-dione | 33.115 | 0.790 | 5.192 |
|  | 7-(beta-Xylosyl)cephalomannine_qt | 38.327 | 0.286 | 5.966 |
|  | Daturilin | 50.365 | 0.768 | 5.732 |
|  | glycitein | 50.479 | 0.238 | 16.320 |
|  | Spinoside A | 39.967 | 0.403 | 8.245 |
|  | (8S,9S,10R,13R,14S,17R)-17-[(E,2R,5S)-5-ethyl-6-methylhept-3-en-2-yl]-10,13-dimethyl-1,2,4,7,8,9,11,12,14,15,16,17-dodecahydrocyclopenta[a]phenanthren-3-one | 45.405 | 0.762 | 5.648 |
|  | 11-Hydroxyrankinidine | 40.003 | 0.662 | 10.800 |
| Pseudostellariae Radix | acacetin | 34.974 | 0.241 | 17.248 |
|  | Linarin | 39.844 | 0.709 | 16.068 |
|  | beta-sitosterol | 36.914 | 0.751 | 5.355 |
|  | luteolin | 36.163 | 0.246 | 15.944 |
|  | Schottenol | 37.423 | 0.751 | 5.633 |
|  | 1-Monolinolein | 37.177 | 0.302 | 4.358 |
| Astragali Radix | Mairin | 55.377 | 0.776 | 8.874 |
|  | Jaranol | 50.829 | 0.291 | 15.501 |
|  | hederagenin | 36.914 | 0.751 | 5.348 |
|  | (3S,8S,9S,10R,13R,14S,17R)-10,13-dimethyl-17-[(2R,5S)-5-propan-2-yloctan-2-yl]-2,3,4,7,8,9,11,12,14,15,16,17-dodecahydro-1H-cyclopenta[a]phenanthren-3-ol | 36.228 | 0.783 | 5.218 |
|  | isorhamnetin | 49.604 | 0.306 | 14.339 |
|  | 3,9-di-O-methylnissolin | 53.742 | 0.476 | 8.996 |
|  | 9,10-dimethoxypterocarpan-3-O-β-D-glucoside | 36.737 | 0.924 | 13.063 |
|  | (6aR,11aR)-9,10-dimethoxy-6a,11a-dihydro-6H-benzofurano[3,2-c]chromen-3-ol | 64.255 | 0.425 | 8.494 |
|  | Bifendate | 31.098 | 0.666 | 17.962 |
|  | formononetin | 69.674 | 0.212 | 17.037 |
|  | isoflavanone | 109.987 | 0.296 | 15.507 |
|  | Calycosin | 47.752 | 0.243 | 17.097 |
|  | kaempferol | 41.882 | 0.241 | 14.743 |
|  | FA | 68.960 | 0.706 | 24.811 |
|  | 1,7-Dihydroxy-3,9-dimethoxy pterocarpene | 39.045 | 0.479 | 7.946 |
|  | quercetin | 46.433 | 0.275 | 14.401 |
| Atractylodis Macrocephalae Rhizoma | 12-senecioyl-2E,8E,10E-atractylentriol | 62.396 | 0.223 | 6.074 |
|  | 14-acetyl-12-senecioyl-2E,8E,10E-atractylentriol | 60.313 | 0.305 | 5.316 |
|  | 14-acetyl-12-senecioyl-2E,8Z,10E-atractylentriol | 63.371 | 0.300 | 6.429 |
|  | (3S,8S,9S,10R,13R,14S,17R)-10,13-dimethyl-17-[(2R,5S)-5-propan-2-yloctan-2-yl]-2,3,4,7,8,9,11,12,14,15,16,17-dodecahydro-1H-cyclopenta[a]phenanthren-3-ol | 36.228 | 0.783 | 5.218 |
|  | 8β-ethoxy atractylenolide Ⅲ | 35.951 | 0.211 | 8.341 |
| Dioscoreae Rhizoma | piperlonguminine | 30.711 | 0.180 | 8.656 |
|  | (-)-taxifolin | 60.506 | 0.273 | 14.373 |
|  | Denudatin B | 61.472 | 0.378 | 7.713 |
|  | Kadsurenone | 54.723 | 0.378 | 9.156 |
|  | hancinol | 64.013 | 0.373 | 4.057 |
|  | hancinone C | 59.046 | 0.390 | 4.143 |
|  | 24-Methylcholest-5-enyl-3belta-O-glucopyranoside_qt | 37.577 | 0.717 | 4.906 |
|  | campesterol | 37.577 | 0.715 | 4.832 |
|  | Isofucosterol | 43.776 | 0.758 | 5.181 |
|  | Stigmasterol | 43.830 | 0.757 | 5.575 |
|  | Dioscoreside C_qt | 36.382 | 0.871 | 5.490 |
|  | diosgenin | 80.878 | 0.810 | 4.137 |
|  | Doradexanthin | 38.156 | 0.537 | 4.130 |
|  | Methylcimicifugoside_qt | 31.693 | 0.237 | 11.293 |
|  | AIDS180907 | 45.328 | 0.773 | 14.863 |
|  | CLR | 37.874 | 0.677 | 4.519 |
| Glycyrrhizae radix et rhizoma | Inermine | 75.183 | 0.538 | 11.717 |
|  | DFV | 32.763 | 0.183 | 17.886 |
|  | Mairin | 55.377 | 0.776 | 8.874 |
|  | Glycyrol | 90.776 | 0.668 | 9.850 |
|  | Jaranol | 50.829 | 0.291 | 15.501 |
|  | Medicarpin | 49.220 | 0.335 | 8.457 |
|  | isorhamnetin | 49.604 | 0.306 | 14.339 |
|  | sitosterol | 36.914 | 0.751 | 5.371 |
|  | Lupiwighteone | 51.636 | 0.367 | 15.630 |
|  | 7-Methoxy-2-methyl isoflavone | 42.565 | 0.199 | 16.887 |
|  | formononetin | 69.674 | 0.212 | 17.037 |
|  | Calycosin | 47.752 | 0.243 | 17.097 |
|  | kaempferol | 41.882 | 0.241 | 14.743 |
|  | naringenin | 59.294 | 0.211 | 16.977 |
|  | (2S)-2-[4-hydroxy-3-(3-methylbut-2-enyl)phenyl]-8,8-dimethyl-2,3-dihydropyrano[2,3-f]chromen-4-one | 31.787 | 0.724 | 14.824 |
|  | euchrenone | 30.287 | 0.574 | 15.889 |
|  | glyasperin B | 65.224 | 0.439 | 16.097 |
|  | glyasperin F | 75.837 | 0.535 | 15.636 |
|  | Isotrifoliol | 31.945 | 0.424 | 7.908 |
|  | (E)-1-(2,4-dihydroxyphenyl)-3-(2,2-dimethylchromen-6-yl)prop-2-en-1-one | 39.617 | 0.351 | 16.165 |
|  | (2S)-6-(2,4-dihydroxyphenyl)-2-(2-hydroxypropan-2-yl)-4-methoxy-2,3-dihydrofuro[3,2-g]chromen-7-one | 60.250 | 0.634 | 4.313 |
|  | Semilicoisoflavone B | 48.778 | 0.547 | 17.021 |
|  | Glepidotin A | 44.722 | 0.347 | 16.094 |
|  | Glepidotin B | 64.463 | 0.345 | 15.980 |
|  | Glypallichalcone | 61.597 | 0.190 | 17.014 |
|  | 8-(6-hydroxy-2-benzofuranyl)-2,2-dimethyl-5-chromenol | 58.437 | 0.381 | 8.714 |
|  | Licochalcone B | 76.757 | 0.194 | 17.018 |
|  | licochalcone G | 49.255 | 0.323 | 15.754 |
|  | Licoricone | 63.578 | 0.471 | 16.369 |
|  | Gancaonin A | 51.075 | 0.404 | 16.821 |
|  | Gancaonin B | 48.794 | 0.449 | 16.487 |
|  | licorice glycoside E | 32.887 | 0.272 | 25.392 |
|  | 3-(3,4-dihydroxyphenyl)-5,7-dihydroxy-8-(3-methylbut-2-enyl)chromone | 66.371 | 0.414 | 15.812 |
|  | 5,7-dihydroxy-3-(4-methoxyphenyl)-8-(3-methylbut-2-enyl)chromone | 30.489 | 0.410 | 14.989 |
|  | 2-(3,4-dihydroxyphenyl)-5,7-dihydroxy-6-(3-methylbut-2-enyl)chromone | 44.152 | 0.415 | 16.770 |
|  | Licocoumarone | 33.211 | 0.357 | 9.655 |
|  | Licoisoflavone | 41.610 | 0.416 | 16.091 |
|  | Licoisoflavone B | 38.929 | 0.547 | 15.732 |
|  | licoisoflavanone | 52.466 | 0.545 | 15.673 |
|  | shinpterocarpin | 80.295 | 0.727 | 6.504 |
|  | (E)-3-[3,4-dihydroxy-5-(3-methylbut-2-enyl)phenyl]-1-(2,4-dihydroxyphenyl)prop-2-en-1-one | 46.268 | 0.306 | 15.236 |
|  | liquiritin | 65.690 | 0.739 | 17.963 |
|  | Glyzaglabrin | 61.069 | 0.353 | 21.202 |
|  | Glabranin | 52.896 | 0.312 | 16.243 |
|  | Glabrone | 52.512 | 0.496 | 16.091 |
|  | 1,3-dihydroxy-9-methoxy-6-benzofurano[3,2-c]chromenone | 48.142 | 0.428 | 8.865 |
|  | 1,3-dihydroxy-8,9-dimethoxy-6-benzofurano[3,2-c]chromenone | 62.901 | 0.528 | 9.322 |
|  | Eurycarpin A | 43.277 | 0.374 | 17.096 |
|  | glycyroside | 37.250 | 0.792 | 14.622 |
|  | (-)-Medicocarpin | 40.994 | 0.951 | 13.196 |
|  | Sigmoidin-B | 34.881 | 0.415 | 14.495 |
|  | (2R)-7-hydroxy-2-(4-hydroxyphenyl)chroman-4-one | 71.123 | 0.183 | 18.093 |
|  | (2S)-7-hydroxy-2-(4-hydroxyphenyl)-8-(3-methylbut-2-enyl)chroman-4-one | 36.565 | 0.323 | 17.947 |
|  | Isoglycyrol | 44.699 | 0.838 | 6.691 |
|  | Isolicoflavonol | 45.170 | 0.419 | 15.553 |
|  | HMO | 38.365 | 0.211 | 16.559 |
|  | 1-Methoxyphaseollidin | 69.981 | 0.637 | 9.526 |
|  | Quercetin der. | 46.449 | 0.334 | 16.612 |
|  | licochalcone a | 40.790 | 0.285 | 16.197 |
|  | icos-5-enoic acid | 30.703 | 0.197 | 5.280 |
|  | Kanzonol F | 32.468 | 0.894 | 9.976 |
|  | 6-prenylated eriodictyol | 39.224 | 0.413 | 16.517 |
|  | 7-Acetoxy-2-methylisoflavone | 38.923 | 0.262 | 17.485 |
|  | 8-prenylated eriodictyol | 53.795 | 0.404 | 15.702 |
|  | gadelaidic acid | 30.703 | 0.197 | 5.248 |
|  | Gancaonin G | 60.435 | 0.394 | 16.135 |
|  | Gancaonin H | 50.104 | 0.784 | 16.636 |
|  | Licoagrocarpin | 58.814 | 0.585 | 9.446 |
|  | Glyasperins M | 72.671 | 0.593 | 15.574 |
|  | Glycyrrhiza flavonol A | 41.275 | 0.595 | 13.708 |
|  | Licoagroisoflavone | 57.282 | 0.487 | 19.642 |
|  | 18α-hydroxyglycyrrhetic acid | 41.161 | 0.709 | 4.960 |
|  | Odoratin | 49.948 | 0.305 | 16.348 |
|  | Phaseol | 78.766 | 0.579 | 9.643 |
|  | Xambioona | 54.849 | 0.874 | 14.499 |
|  | quercetin | 46.433 | 0.275 | 14.401 |
