# Supplementary material for: Impact of Qi-Invigorating Traditional Chinese Medicines on Diffuse Large B Cell Lymphoma Based on Network Pharmacology and Experimental Validation
Source: Front Pharmacol. 2021 Dec 9;12:787816. doi: 10.3389/fphar.2021.787816 (PMC8699731; doi:10.3389/fphar.2021.787816)
Supplement: Supplementary file 2 [file Table2.docx]

Supplement Table 2. The target names of Qi-invigorating herbs

| Herbs | Targets |
| --- | --- |
| Ginseng Radix et Rhizoma | CYP1B1 NR1H3 AHR ALOX5 UGT1A8 SREBF2 CYP7A1 ALOX12 CDK1 UGT1A7 TRPV4 RPS6KA3 KIAA0101 ABCA1 IL10 PTGS1 DHCR24 BCL2A1 SREBF1 HRH1 ABCG5 ICAM1 TBXA2R NR1I2 CASP3 UGT3A1 ABCG8 PRKCA UGT1A3 F2 WDTC1 ABCB11 APOE PPIG PLA2G1B PTGS2 IL8 SLCO1B1 UGT1A9 TNF CA2 PPARD FABP4 NPC1L1 ABCC1 XDH ACHE TYR PPARG CA4 HSD17B1 NOX4 ABCB1 ESRRA PPARA HSD17B2 FLT3 ABCG2 CA12 CA7 ADORA1 AR AKR1B1 |
| Panacis Quinquefolii Radix | PDE5A PDE10A PDE4D OPRD1 SREBF2 CYP7A1 CASP3 ABCG8 ABCB11 OPRL1 APOE OPRK1 TAS2R30 DHCR24 SREBF1 BCL2A1 KNG1 ABCG5 ICAM1 OPRM1 SST PDE3B PDE3A KCNH2 CYP2C19 AR PDE2A |
| Codonopsis Radix | NR1H3 MMP9 MMP13 SREBF2 CDK4 FOS EGFR MAPK8 AKT1 SMAD2 ABCA1 IL10 SP1 ABCG5 CDK2 MAPK1 JUN CASP3 ABCG8 MAPK3 CCNA2 IL8 SLCO1B1 TNF TNKS2 CA2 SYK MMP12 CTSD MMP2 AKR1B10 CYP1B1 APP NPC1L1 PTPRS XDH GSK3B CD38 CCNB3 CDK1 CCNB1 CCNB2 CA4 NOX4 TNKS CDK5R1 CDK5 FLT3 MAOA ABCG2 RORC CA12 CA7 ADORA1 ALOX5 PARP1 TOP1 TTR AR GLO1 AKR1B1 ARG1 |
| Pseudostellariae Radix | CYP1B1 MMP9 CYP1A2 SREBF2 VEGFA CYP7A1 IL5 IL13 FOS EGFR MAPK8 SMAD2 AKT1 DHCR24 SREBF1 ABCG5 CDK2 ICAM1 SELE CYP1A1 JUN NR1I2 CASP3 ABCG8 ABCB11 CCNA2 APOE STAT1 TNKS2 CA2 SYK MMP12 MMP2 AKR1B10 APP PTPRS NPC1L1 XDH GSK3B CD38 CCNB3 CDK1 CCNB1 CCNB2 CA4 NOX4 NR1H3 TNKS CDK5R1 CDK5 FLT3 MAOA ABCG2 CA12 CA7 ADORA1 ALOX5 PARP1 TOP1 TTR GLO1 AKR1B1 ARG1 |
| Astragali Radix | CDK1 AKT1 CYP1B1 ABCC1 AHR PIM1 CA2 BACE1 HMGCR CA3 AKR1C3 MMP2 AKR1B10 AURKB CSNK2A1 PTK2 TYMS NUAK1 PIK3R1 XDH ACHE GSK3B TYR CA14 CA4 DAPK1 HSD17B1 ALOX15 DRD4 CYP19A1 PYGL HSD17B2 KDR F2 CA9 ABCG2 CA12 AKR1C2 PLA2G1B AKR1A1 CYP51A1 GLO1 NEK2 CA13 CAMK2B POLB MMP13 PLK1 ALOX12 EGFR ADORA2A IGF1R SRC PKN1 GPR35 MMP3 MET NOX4 AKR1C1 CA1 ABCB1 AXL ESRRA AVPR2 AKR1C4 CA5A SAE1 UBA2 FLT3 MAOA ALK MPO CA7 ADORA1 ALOX5 CA6 NEK6 MMP9 AR IL2 CXCR1 AKR1B1 UGT1A8 HCK PNLIP UGT1A7 SLC2A2 UGT1A10 CYP2C8 MCL1 NR1I2 UGT2B7 PRKCD UGT1A3 STK17B CYCS HMOX1 NOS3 HIBCH ATP5B NOS2 PON2 PDE4A BIRC5 BLVRB MAPK9 TOP2A RPS6KA3 MAPK8 CASP9 SP1 LMNB1 CYP1A1 UGT3A1 CASP3 MAPK3 UGT1A1 HMGB1 CASP8 TOP1 UGT1A9 |
| Dioscoreae Rhizoma | NR1H3 NPC1L1 RORC ABCG1 HMGCR SREBF2 CETP CYP7A1 ABCG8 APOA1 PTAFR LCAT ABCA1 HSD3B2 APOB IL10 DHCR24 CYP11A1 ABCG5 IL8 SLCO1B1 TNF DHCR7 |
| Glycyrrhizae Radix et Rhizoma | CDK1 AKT1 CYP1B1 ABCC1 ABCB1 AHR PIM1 CA3 AKR1C3 MMP2 AURKB PTK2 SHBG NUAK1 XDH TYR HSD17B1 CYP19A1 HSD17B2 KDR ABCG2 AKR1A1 GLO1 CA13 CAMK2B MMP13 ALOX12 ADORA2A IGF1R GPR35 MMP3 MET AKR1C1 CA1 AXL ESRRA AVPR2 AKR1C4 SAE1 UBA2 MPO ALOX5 CA6 CXCR1 AKR1B1 CA2 BACE1 AKR1B10 CSNK2A1 PIK3R1 ACHE GSK3B CA14 CA4 DAPK1 ALOX15 DRD4 PYGL F2 CA9 CA12 AKR1C2 PLA2G1B NEK2 POLB PLK1 NPC1L1 EGFR PKN1 SRC NOX4 CA5A FLT3 MAOA ALK CA7 CBR1 ADORA1 MMP9 NEK6 IL2 BDNF HCK PNLIP UGT1A7 UGT1A10 DHCR24 SREBF1 CYP2C8 NR1I2 CYCS HMOX1 ATP5B NOS2 RAPGEF1 SREBF2 TOP2A MAPK8 CASP9 ICAM1 LMNB1 CYP1A1 LDLR PPARA UGT3A1 MAPK3 UGT1A1 APOB CASP8 UGT1A9 CYP1A2 UGT1A8 SLC2A2 MCL1 CCL2 ABCG8 UGT2B7 UGT1A3 PRKCD ABCB11 STK17B NOS3 HIBCH PDE4A PON2 BIRC5 CYP7A1 BLVRB MAPK9 RPS6KA3 SP1 ABCG5 CASP3 HMGB1 APOE TOP1 |
